# Plasmid-encoded toxin defence mediates mutualistic microbial interactions

**DOI:** 10.1038/s41564-023-01521-9

**Published:** 2023-12-27

**Authors:** Sarah Moraïs, Michael Mazor, Omar Tovar-Herrera, Tamar Zehavi, Alvah Zorea, Morya Ifrach, David Bogumil, Alexander Brandis, Jens Walter, Natalie Elia, Eyal Gur, Itzhak Mizrahi

**Affiliations:** 1 National Institute of Biotechnology in the Negev, Ben-Gurion University of the Negev, Be’er Sheva, Israel; 2 Department of Life Sciences, Ben-Gurion University of the Negev, Be’er Sheva, Israel; 3 The Goldman Sonnenfeldt School of Sustainability and Climate Change, Ben-Gurion University of the Negev, Be’er Sheva, Israel; 4 Life Sciences Core Facilities, Weizmann Institute of Science, Rehovot, Israel; 5 Department of Medicine, University College Cork, Cork, Ireland

**Keywords:** Microbial ecology, Bacterial toxins

## Abstract

Gut environments harbour dense microbial ecosystems in which plasmids are widely distributed. Plasmids facilitate the exchange of genetic material among microorganisms while enabling the transfer of a diverse array of accessory functions. However, their precise impact on microbial community composition and function remains largely unexplored. Here we identify a prevalent bacterial toxin and a plasmid-encoded resistance mechanism that mediates the interaction between Lactobacilli and Enterococci. This plasmid is widespread across ecosystems, including the rumen and human gut microbiota. Biochemical characterization of the plasmid revealed a defence mechanism against reuterin, a toxin produced by various gut microbes, such as *Limosilactobacillus reuteri*. Using a targeted metabolomic approach, we find reuterin to be prevalent across rumen ecosystems with impacts on microbial community structure. *Enterococcus* strains carrying the protective plasmid were isolated and their interactions with *L. reuteri*, the toxin producer, were studied in vitro. Interestingly, we found that by conferring resistance against reuterin, the plasmid mediates metabolic exchange between the defending and the attacking microbial species, resulting in a beneficial relationship or mutualism. Hence, we reveal here an ecological role for a plasmid-coded defence system in mediating a beneficial interaction.

## Abstract (web summary)

*Enterococcus* strains harbour a plasmid-encoded defence against reuterin, a toxin produced by *Limosilactobacillus reuteri*, mediating a mutualistic metabolic interaction between these two gut microbiota members.

## Main

Plasmids are circular self-replicating double-stranded DNA molecules that reside within bacterial hosts^1^. Plasmids have been recognized as key vectors of genetic exchange between microbial chromosomes. Their high abundance in microbial populations sampled from various habitats indicates that they have an important ecological role^2^. Despite their apparent great importance in nature, the role of plasmids within microbial communities is poorly understood, as they are usually studied as individual entities within specific hosts, neglecting the environmental and community context.

Plasmids are composed of a conserved DNA backbone that includes replication and mobilization genes, which are important for plasmid maintenance within their specific host and transfer among other microbial hosts. They also carry a variable assortment of accessory genes, which often contribute to the phenotypic diversity of their host. Plasmids isolated from different environments encode a versatile array of accessory functions, ranging from antibiotic resistance to nitrogen fixation^3,4^. These plasmid-borne functions may confer an advantage upon their host in its local environment, making the burden of carrying the plasmid worthwhile to the host organism^2,5–9^.

Knowledge of the biology of plasmids and the functions that are mobilized by them can provide insights into the genetic interactions and the ecological roles played by plasmids in individual microbial species and complex microbial communities. Although generally studied in individual species, the role of plasmids can be even greater in dense microbial communities where close proximity exists among microbial individuals of different lineages^10^. In such dense communities, the ecological role of plasmids as accessory gene carriers can be instrumental to niche expansion, as they allow acquisition of potential functions relevant to resource utilization, as well as weapons and defence mechanisms, thereby expanding the niche space^10,11^.

The rumen microbial ecosystem is one of the most studied complex microbial environments due to its importance to agriculture, potential for comprehending fibre-degradation principles, contribution to carbon cycling and bioenergy, and its link to environmental issues, notably the greenhouse effect^12,13^. This complex microbial community is composed of a multitude of different microbial species co-residing at a density greater than 10^11^ ml^−1^, thus rendering this ecosystem ideal for studying the role of plasmids in microbial community ecology. We focused on this system to gain further insights into the ecological role of plasmids in their natural environments. To this end, we identified prevalent natural plasmid candidates with the underlying assumption that plasmids with high occupancy encoding for metabolic accessory genes could provide insights to elucidate plasmid-mediated ecology.

In our previous explorations of the plasmidome^14,15^, we have reported that the mobile genetic pool carries highly diverse accessory genes with potentially high ecological relevance. In the current study, we explored accessory rumen plasmidome genes for recurring functions with high occupancy across multiple ecosystems and their importance within them. Our findings reveal the widespread occurrence of a plasmid accessory function in the rumen and human gut systems that provides resistance to a toxin and facilitates a mutualistic relationship between toxin-producing and plasmid-carrying defending microbes, suggesting a pivotal role for plasmids in orchestrating higher-order microbial interactions.

## Results

### Enterococcal plasmid-borne 1,3-propanediol dehydrogenase (1,3-PD) is widespread in the rumen

We have previously identified many unconventional accessory functions that are carried on plasmids within the environment^14,15^. Here we aimed to explore the potential ecological role of a plasmid encoding one such putative function, the 1,3-PD gene that we found to be carried in high abundance within the rumen plasmidome^14,15^. This putative function was found to be coded on plasmids coming from 11 samples out of 78 metagenomes from our previous study^16^. These metagenome-assembled rumen plasmids were assembled via Recycler algorithm^17^ and extracted from metagenomic reads or sequencing of extracted plasmidomes, as described in previous studies^15,18,19^. This finding raised the hypothesis that this putative gene could have high ecological relevance, as it occurs repeatedly in the mobile genetic pool. In all cases, the putative (1,3-PD) gene was identified in a 5,326-bp plasmid that did not contain any mobilization genes coding for plasmid transmissibility (Supplementary Table 1). This suggests that the plasmid is constrained to its bacterial hosts or transferred by a non-conjugative mechanism^20^. The plasmid contains additional putative genes, annotated as recombinase, integrase, transposase and antiterminator functions.

**Fig. 1:**
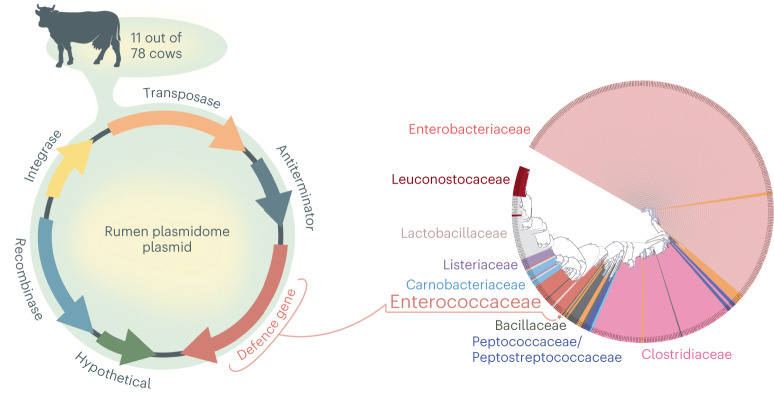
Identification of rumen plasmid with potential ecological relevance from the rumen plasmidome. Left: plasmid map of 1,3-PD plasmid retrieved from the rumen microbiome of a 78-animal cohort. Right: phylogenetic tree of 1,3-PD genes retrieved by PhyloGenie^21^ from the NR NCBI database. Bacillaceae, Clostridiaceae, Enterobacteriaceae, Enterococcaceae, Lactobacillaceae, Leuconostocaceae and Listeriaceae families are colour coded and appear on the tree. Proteins assigned to Carnobacteriaceae family are labelled in light blue, Peptococcaceae/Peptostreptococcaceae in darker blue and other phylogenies in light orange (including the Planococcaceae, Pseudomonadaceae, Thermoactinomycetaceae, Yersiniaceae, Fusobacteriaceae, Moraxellaceae and Morganellaceae families). A red star and a red square mark the position of the 1,3-PD rumen plasmidome gene. Bootstrapping higher than 0.9 is indicated by a blue circle. Source data

We next applied a gene-tree approach to determine the phylogeny of the plasmidic 1,3-PD gene by retrieving homologue genes from the NR NCBI database and phylogenetically classifying the 1,3-PD genes using the PhyloGenie pipeline^21^. This analysis positioned the plasmid coding the 1,3-PD gene within the Enterococcaceae family clade and suggested that its members represented the host microbes of these plasmids (Fig. 1).

### 1,3-PD plasmid is suspected to protect against reuterin toxin

**Fig. 2:**
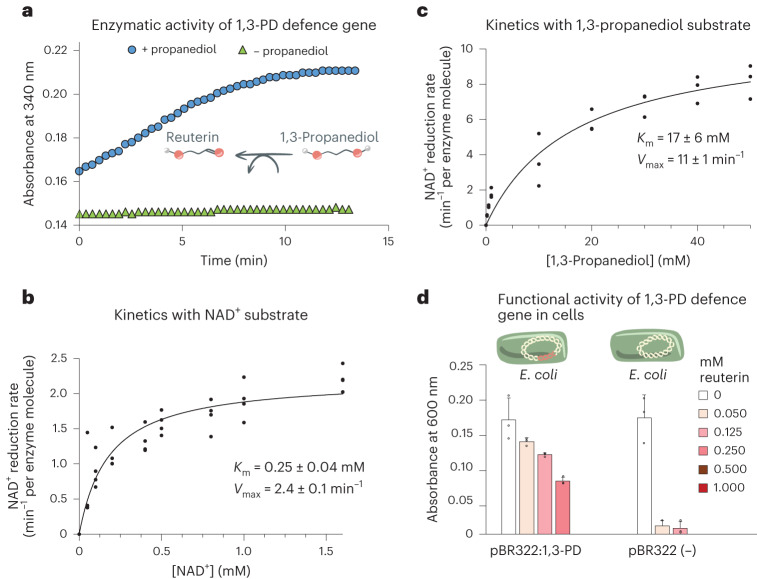
Biochemical characterization of the 1,3-PD defence gene and its functional activity within a bacterial host. **a**, Enzymatic activity of the 1,3-PD enzyme over time, using 1,3-propanediol as a substrate. **b**,**c**, Michaelis–Menten analysis of 1,3-PD activity at increasing concentrations of either NAD^+^ (**b**) or 1,3-propanediol (**c**). The measurements were carried out in technical triplicates, for which actual data are presented. The solid curves were generated by fitting the Michaelis–Menten equation to the data, assuming non-cooperative binding of NAD^+^ and 1,3-propanediol by the enzyme. **d**, Protective effect against reuterin of 1,3-PD expression in *E. coli*. ER2566 strains constitutively expressing 1,3-PD from plasmid pBR322 were grown in increasing concentrations of reuterin (0 to 1 mM final concentration) in 200 μl of LB medium (volumes of cultures were homogenized across samples using sterile water). Following 24 h at 37 °C, turbidity was measured at 600 nm. Control strains carried an empty vector. The measurements were carried out in biological triplicates, for which actual data, averages and standard deviations are presented. Source data

The 1,3-PD enzymes were reported to be part of a chromosomal operon coding for glycerol fermentation to propionate^22^. The fact that only the 1,3-PD putative gene was coded on the rumen plasmid suggested that it might pose a different ecological role from that of glycerol fermentation. Studies have shown that genes carried on plasmids can be expressed at higher levels compared with their chromosomal counterparts. This higher expression could confer a selective advantage to bacteria carrying the plasmid with the defence gene, allowing them to better compete with other bacterial species for resources in their environment^23^. A literature search revealed that the 3-hydroxypropionaldehyde molecule, also referred to as reuterin, has antimicrobial activity and is often produced by gut microbes, notably *Limosilactobacillus reuteri* (Fig. 2a inset)^24,25^. Thus, we considered the possibility that the putative rumen plasmid gene might function as a defence mechanism against the antibacterial activity of reuterin. A basic local alignment search tool (Blast) against the NR database of the putative rumen plasmid 1,3-PD gene variant revealed 74% amino-acid sequence identity and 82% similarity to the previously characterized 1,3-PD from *Clostridium butyricum*^22^ and showed high similarities with other previously characterized enzymes from this family (Extended Data Fig. 1)^22,26–28^. This further suggested that this putative plasmid-coded enzyme converts reuterin into 1,3-propanediol and therefore detoxifies it.

### 1,3-PD gene is active in vitro and detoxifies antibacterial reuterin

**Extended Data Fig. 2:**
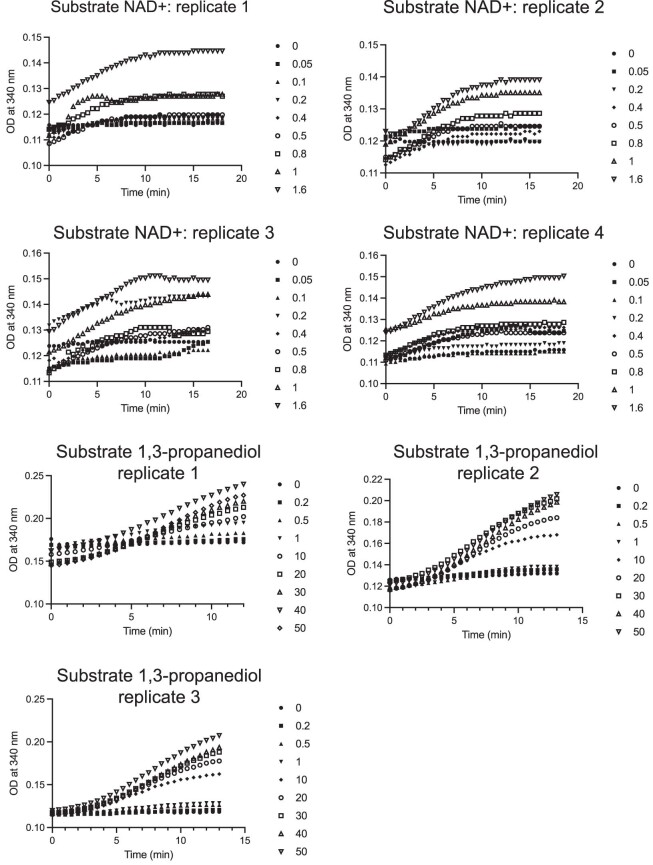
Measurements of 1–3 PD kinetics. Each graph depicts a representative replicate of the absorbance at 340 nm measured over time at the indicated concentrations of NAD+ or 1,3-PD at increasing concentrations. Source data

We tested whether 1,3-PD can detoxify reuterin, by cloning the gene and overexpressing the enzyme in *E. coli*. The purified enzyme was clearly found to be active via measurement of oxidation by NAD^+^ of, rather than reuterin, the commercially available 1,3-propanediol, by monitoring the absorbance of the formed NADH at 340 nm (Fig. 2a). Next, we sought to assess the enzyme’s affinity to its substrates. Accordingly, titration experiments were carried out, where NAD^+^ reduction rates were measured at increasing concentrations of NAD^+^ or 1,3-propanediol. The results yielded classic Michaelis–Menten curves, with a *K*_m_ of 0.25 ± 0.04 mM for NAD^+^ (Fig. 2b and Extended Data Fig. 2) and a *K*_m_ of 17 ± 6 mM (Fig. 2c and Extended Data Fig. 2) for 1,3-propanediol. As 1,3-propanediol titrations were carried out with NAD^+^ concentrations that were close to saturation, the resulting *V*_max_ value (11 ± 1 min^−1^ per enzyme molecule) extracted from the resulting data (Fig. 2c) closely corresponded to the enzyme catalytic rate constant (*k*_cat_). These kinetic parameters closely resemble the values previously reported for the homologous enzyme from *Klebsiella pneumoniae*^27,29^, suggesting that reuterin inactivation might be the role of the plasmid-coded enzyme in the rumen.

### Plasmid-encoded 1,3-PD protects bacterial cells from reuterin toxin

As the plasmid-coded 1,3-PD defence gene was not part of a full operon but a standalone gene on the plasmid, we next tested whether it would confer reuterin resistance in bacterial cells. To this end, we cloned the gene into an *E. coli* plasmid pBR322 under the beta-lactamase promoter and, following transformation, grew the bacteria at increasing concentrations of reuterin (0 to 1 mM final concentration) compared to the control. The results of this experiment showed that in all reuterin concentrations, the strain carrying the 1,3-PD defence gene exhibited significantly higher survival rates than the control strain lacking the gene, the latter showing no apparent growth above 0.125 mM reuterin, thus demonstrating the protective effect of the 1,3-PD defence enzyme against reuterin (Fig. 2d). The protective effect of the enzyme decreased at higher reuterin concentrations of 0.5 and 1 mM where no growth of both strains was observed, with an half maximal inhibitory concentration (IC_50_) value between 0.125 mM and 0.25 mM reuterin.

### Reuterin is prevalent across and influences rumen ecosystems

**Fig. 3:**
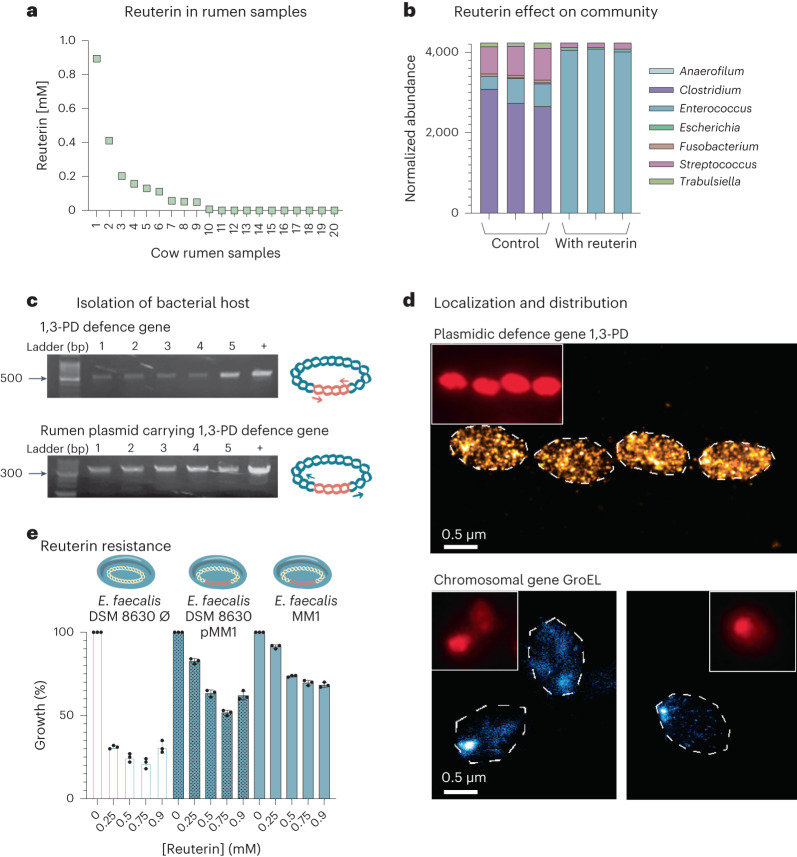
Effect of reuterin on rumen microbial composition and isolation of the bacterial host. **a**, Distribution plot representing the concentration of reuterin (in mM) detected in rumen samples from 20 cows. **b**, Phylogenetic analysis of the cultured microbial rumen community after serial passaging with or without reuterin addition at the genus level. The bar graph represents the normalized abundances of the genera. Genera with low abundances in control communities, including *Adlercreutzia*, *Bacteroides*, *Bifidobacterium*, *Blautia*, *Butyricicoccus*, *Butyrivibrio*, *Dialister*, *Peptococcus*, *Prevotella*, *Roseburia* and *Sediminibacterium*, are not represented. **c**, Top gel: PCR of part of the 1,3-PD gene (expected product length 560 bp), amplified from the plasmid-purified fraction of five isolates. Bottom gel: PCR of the whole plasmid encoding the 1,3-PD gene, using reverse primers (expected product length ~5,300 bp), amplified from the plasmid-purified fraction of five isolates. In both gels, plasmid pBR322:1,3-PD was used as a positive control template. Both gels were reproducible in two distinct experiments. Illustrations on the right represent the plasmid in blue, and the 1,3-PD gene in orange and arrows indicate the primers used for the PCR. **d**, STORM and wide-field (inset) imaging of *E. faecalis* MM1 cells labelled by CARD-FISH with specific probes for the 1,3-PD defence plasmidic gene (top) or GroEL chromosomal gene (bottom). White dashed lines delimit the bacterial cell walls. Similar images were obtained from two distinct experiments. **e**, Comparative resistance of *E. faecalis* MM1 plasmid-carrying strain and *E. faecalis* DSM 8630 pMM1 transformed with the rumen plasmid with the 1,3-PD defence gene against reuterin and *E. faecalis* DSM 8630 ∅ (with an empty plasmid backbone lacking the 1,3-PD defence gene). The strains were grown at increasing concentrations of reuterin (0 to 0.9 mM) in 1,000 μl basal defined medium and incubated for 24 h at 37 °C (volumes of cultures were homogenized across samples using sterile water). Turbidity measurements at 600 nm were used to determine the protection of the 1,3-PD defence gene against reuterin. The *y* axis represents the % growth of a specific strain as compared to growth of the same strain without reuterin. The measurements were carried out in biological triplicates, for which actual data, averages and standard deviations are presented. Source data

As our findings show that reuterin has an effect on bacterial cells and the plasmid carrying the 1,3-PD defence gene could confer reuterin resistance in bacterial cells, we first examined whether reuterin is present in natural rumen ecosystems. To this end, we measured reuterin concentrations in 20 bovine rumen samples using an LC–MS-targeted metabolomics approach. Our findings showed that reuterin is indeed present in 10 of the 20 rumen samples in concentrations ranging from 0.07 to 0.89 mM (Fig. 3a).

**Extended Data Fig. 3:**
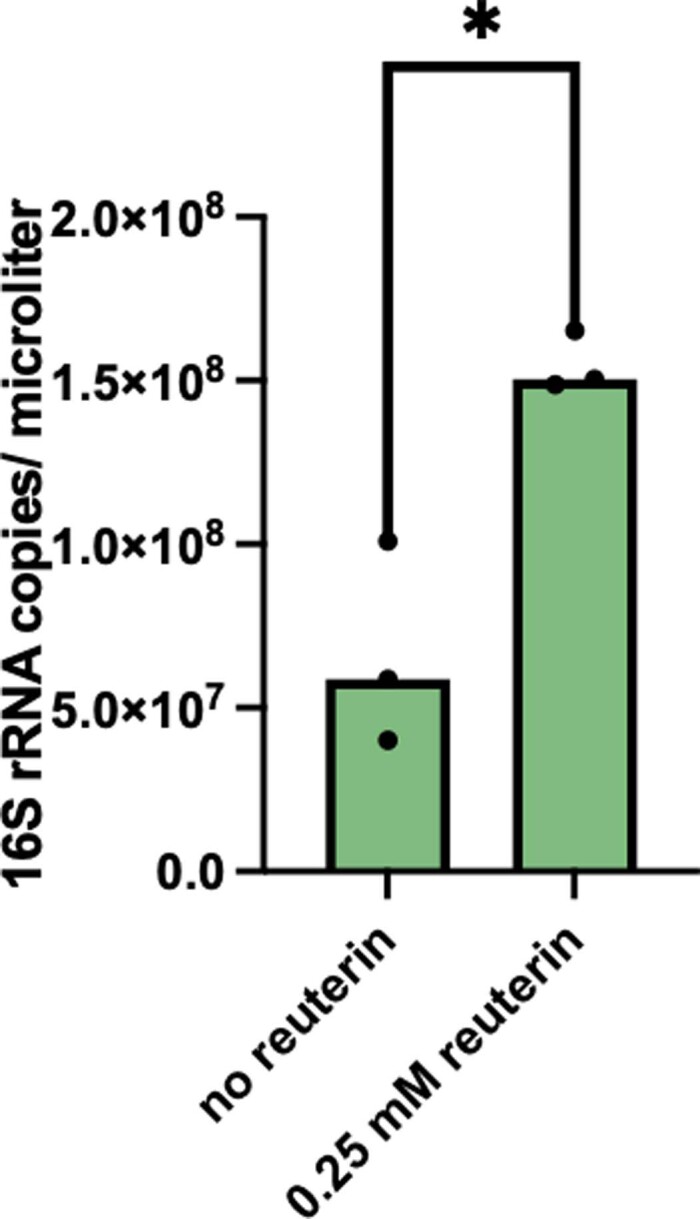
Effect of reuterin on rumen microcosms biomass. The 16S rRNA copies of the microcosms are given for three biological triplicates (actual values are indicated). Asterisk represents *P* value of 0.009 with two-sided t-test without adjustment for multiple comparisons. Source data

To examine the effect of reuterin and the defence system on members of the rumen ecosystem, we exposed to reuterin selected rumen microcosms in which the plasmid was bioinformatically detected. A taxonomic analysis of the 16S ribosomal (r)RNA gene showed a significant increase in the relative abundance of the *Enterococcus* genus (from 7% to 96%) in the reuterin-treated microcosms compared with the more diverse community phenotypes without the addition of reuterin (Fig. 3b); results are in accordance with our bioinformatic analysis for the phylogenetic assignment of the Enterococcaceae family members as microbial hosts of the rumen plasmid containing 1,3-PD defence gene. The broad growth inhibition of reuterin affected many rumen bacteria observed here, aligning with an earlier study demonstrating that reuterin altered the growth of the majority of the tested intestinal bacteria^30^. Moreover, the increase in *Enterococcus* abundance was accompanied by an overall increase in biomass, as measured by total 16S rRNA copy number using qPCR (Extended Data Fig. 3; *t*-test *P* = 0.028). These findings suggest that the reduced competition among a less diverse bacterial community allowed for the proliferation of Enterococci in the reuterin-treated microcosms.

### 1,3-PD plasmid in *E. faecalis* isolates confers reuterin resistance

**Extended Data Fig. 4:**
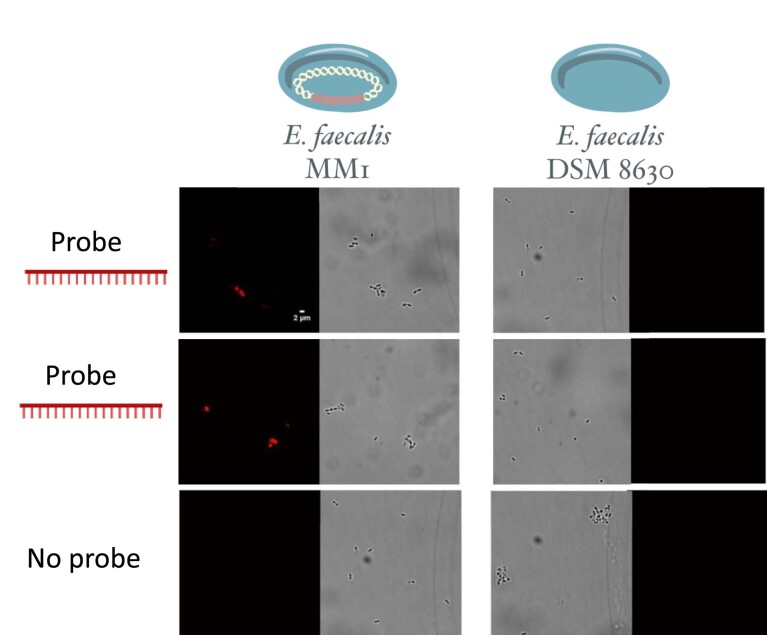
Plasmid labeling of *E. faecalis* strains. CARD-FISH of *E. faecalis* DSM 8630 (as a negative control) and *E. faecalis* MM1 using the specific 1,3-PD probe. Scale bar is indicated. Source data

**Extended Data Fig. 5:**
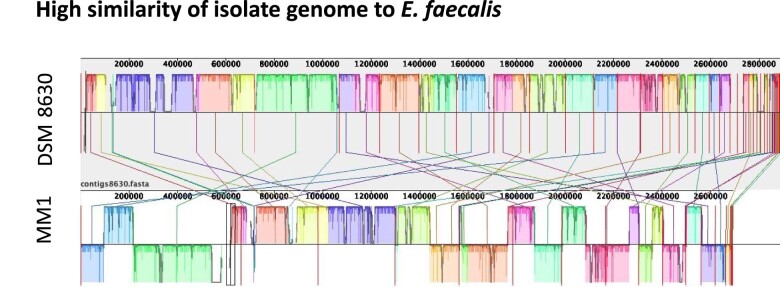
Similarity of the *E. faecalis* strains MM1 and DSM 8630 genomes and predicted proteomes. Similarity of DNA contigs between the DSM 8630 and MM1 strains. Contigs were aligned using the Mauve genome alignment software^67^.

Following our above-described results that revealed an enrichment of the Enterococcaceae family in reuterin-treated rumen samples, we tested whether we could use reuterin to enrich and isolate microbes that carry the plasmid with the 1,3-PD defence gene. The reuterin-resistant enriched microbial community was used for selection on reuterin-containing agar plates. Plates containing high concentrations of reuterin presented an increased number of a specific type of colony morphology. Taxonomic identification of these colonies by 16S rRNA gene Sanger sequencing showed 95% identity to members of the Enterococcaceae family, specifically to *E. faecalis* strains, as predicted by our bioinformatics analysis. We therefore designated the new isolated strain as *E. faecalis* MM1. We also validated the presence of the plasmid carrying the 1,3-PD defence gene in these isolates by applying PCR (Fig. 3c) and a microscopic approach using a catalysed reporter deposition single-molecule fluorescence in situ hybridization (CARD-FISH) probe that targets the 1,3-PD defence gene, which was then visualized by stochastic optical reconstruction microscopy (STORM) (Fig. 3d). Using both approaches, we observed an exclusive and unique signal only for the isolated *E. faecalis* MM1 cells, but not for *E. faecalis* DSM 8630 strain lacking the plasmid (Fig. 3c,d and Extended Data Fig. 4). With the microscopic approach, we observed a scattered localization of signal when the cells were stained with the 1,3-PD-specific probe, as opposed to the confined localization observed using a different probe that targets the GroEL gene that is localized on the chromosome. We then performed whole-genome sequencing of one of the isolated strains together with a DSM strain of *E. faecalis*. We found that the two strains have an average nucleotide identity of 98.87% (ref. ^31^), with an extensive overlap of contigs (Extended Data Fig. 5). We then measured the reuterin resistance of the *E. faecalis* MM1 plasmid-carrying strain against that of *E. faecalis* DSM 8630 to which the pMM1 plasmid was transformed (*E. faecalis* DSM 8630 pMM1) and *E. faecalis* DSM 8630 ∅ to which an empty plasmid backbone was transformed (Fig. 3e). Our results show that the *E. faecalis* MM1 culture and *E. faecalis* DSM 8630 pMM1 exhibited similar higher resistance patterns across the elevated concentrations of reuterin in the medium, as compared with *E. faecalis* DSM 8630 ∅, further supporting the importance of the plasmid in the defence against reuterin.

### Mutualism and metabolic exchange between the two rival species

**Fig. 4:**
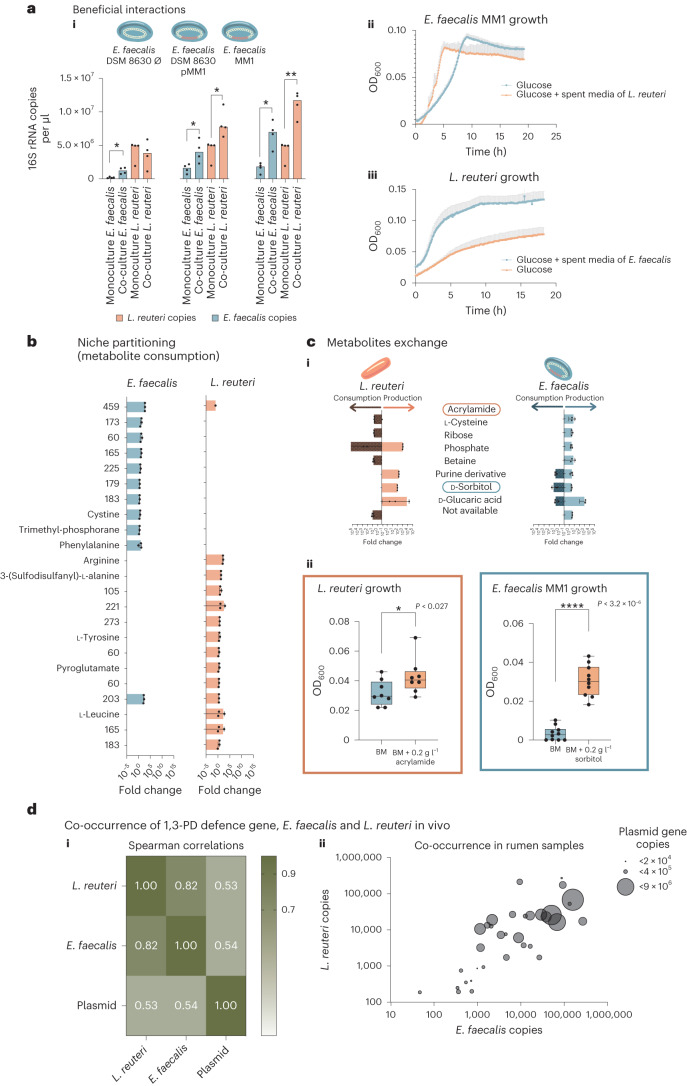
Metabolic interaction between the reuterin producer and degrader. **a**(i), Bar graph representing quantitative PCR analysis (16S rRNA gene) of mono- and co-cultures of *L. reuteri* (orange) with the different *E. faecalis* strains (blue) from 4 biological replicates using specific primers for each strain. Asterisks represent *P* values using two-sided *t*-test (values from left to right: 0.005, 0.027, 0.021, 0.003 and 0.001). (ii),(iii), Growth curves of *L. reuteri* (iii) or *E. faecalis* (ii) as represented by OD measured at 600 nm over time with and without *E. faecalis* or *L. reuteri* spent media. Mean + s.d. **b**, Bar graphs representing the fold change of the metabolites consumed by *L. reuteri* or *E. faecalis* cultured on basal defined medium as analysed by LC–MS. Fold changes of the consumed metabolites compared to the respective blank media are presented. **c**(i), Bar graphs displaying the fold change of the consumed and/or produced metabolites by each bacterial species. The analysis was carried out on samples cultured on spent medium obtained from the two strains on which they were grown reciprocally. All LC–MS measurements (in **b** and **c**) were performed on biological triplicates; actual values (dots), mean ± s.d. are presented. (ii), Boxplots representing supplementation assays with commercial acrylamide (*n* = 8) or sorbitol (*n* = 10) added to basal defined medium as the sole carbon source before growing *L. reuteri* or *E. faecalis*, respectively, for 20 h at 37 °C. All points are displayed and the means are represented as a line. Actual OD_600_ values are displayed; *P* values obtained with two-sided *t*-test. Boxes represent the interquartile range (IQR) central half of the data, the whiskers indicate the range of the data, minimum to maximum values, and individual dots represent all the measurements. **d**, Co-occurence of 1,3-PD gene, *E. faecalis* and *L. reuteri* in vivo. (i), Spearman correlations and (ii) co-occurrence scatterplot between the *L. reuteri*, *E. faecalis* and 1,3-PD gene abundances as determined by quantitative PCR using specific primers on 34 individual rumen samples. The *x* and *y* axes in ii represent the 16S rRNA gene copy number of *E. faecalis* and *L. reuteri*, respectively, and the circle sizes represent the 1,3-PD gene copy number. Source data

**Extended Data Fig. 6:**
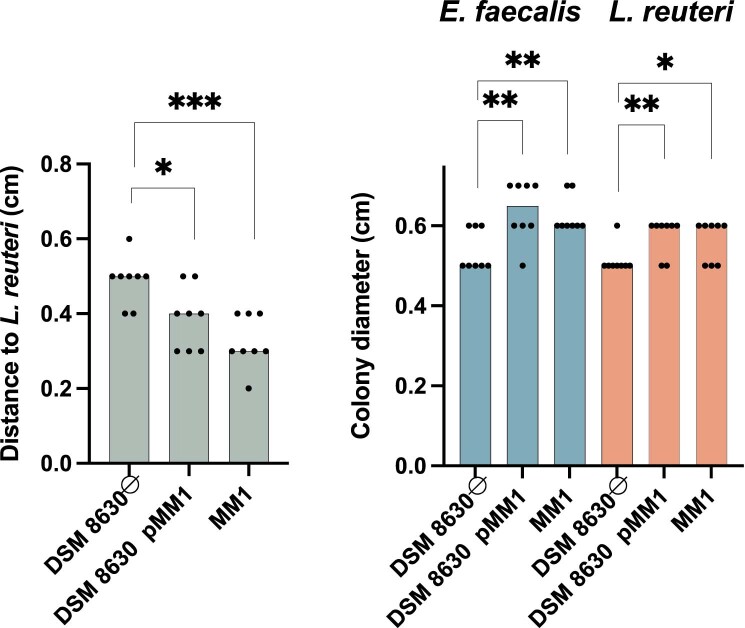
Proximity assay between *L. reuteri* and *E. faecalis* strains. Left panel, the bar graph displays the rounded distance of *E. faecalis* colonies to *L. reuteri* colonies with reuterin in the media. Stars represent statistical significance with two-sided t-test of *P* value 0.017 (*) or 0.00027(***) without adjustment for multiple comparisons. Right panel, the bar graph represents the rounded colonies diameter of *E. faecalis* and *L. reuteri* strains with reuterin in the media. For both panels, the assays were performed in eight biological triplicates for which actual values and median, are presented. Stars represent statistical significance with two-sided t-test of p value <0.01 (**) and <0.05 (*) without adjustment for multiple comparisons (*P* values from left to right, 0.0075, 0.0031, 0.0089 and 0.04). Source data

The high occurrence of the plasmid carrying the 1,3-PD defence gene in the rumen ecosystem and within the *E. faecalis* strain raised the question of whether the bacterial hosts carrying the 1,3-PD defence plasmid benefit from additional advantages other than reuterin resistance. Inspired by the symbiotic relationships that exist in nature^32^, we speculated that a potential benefit for carrying the defence plasmid would be a dependence on close proximity since reuterin is most effective as a weapon when in close proximity. Indeed, when we co-cultured *E. faecalis* and *L. reuteri*, we identified a beneficial effect reflected in an increase in biomass of both species compared with the individual monocultures (Fig. 4a(i)). It is important to consider that in natural environments, microbial growth is often asynchronous^33^ and reuterin may already be present during microbial interactions. Therefore, we externally added reuterin to our experiments to create controlled conditions that closely resemble the rumen environment, where reuterin is prevalent during the growth of both bacterial species as seen in our measurements of the rumen environment and in other gut environments^34–37^. Our results demonstrate that in co-culture, the benefit on *L. reuteri* growth is observed only with *E. faecalis* strains containing 1,3-PD gene on plasmids and the benefit on *E. faecalis* growth is enhanced with the plasmid coding the 1,3-PD gene (Fig. 4a(i)). Proximity assays on solid media further corroborated these results; indeed, we observe an increase in the colony diameter of both *E. faecalis* and *L. reuteri* strains and a reduced distance between the two bacterial colonies only when the plasmidic 1,3-PD gene is present, allowing for increased beneficial interaction between the strains (Extended Data Fig. 6). Furthermore, when we cultured *E. faecalis* MM1 on *L. reuteri* spent growth media, we identified a significant increase in the generation time of *E. faecalis* when exposed to *L. reuteri* metabolites (Fig. 4a(ii)). Surprisingly, *L. reuteri* also showed a remarkable increase in fitness when exposed to *E. faecalis* metabolites (Fig. 4a(iii)), further suggesting that this interaction is dependent on metabolite exchange.

**Fig. 5:**
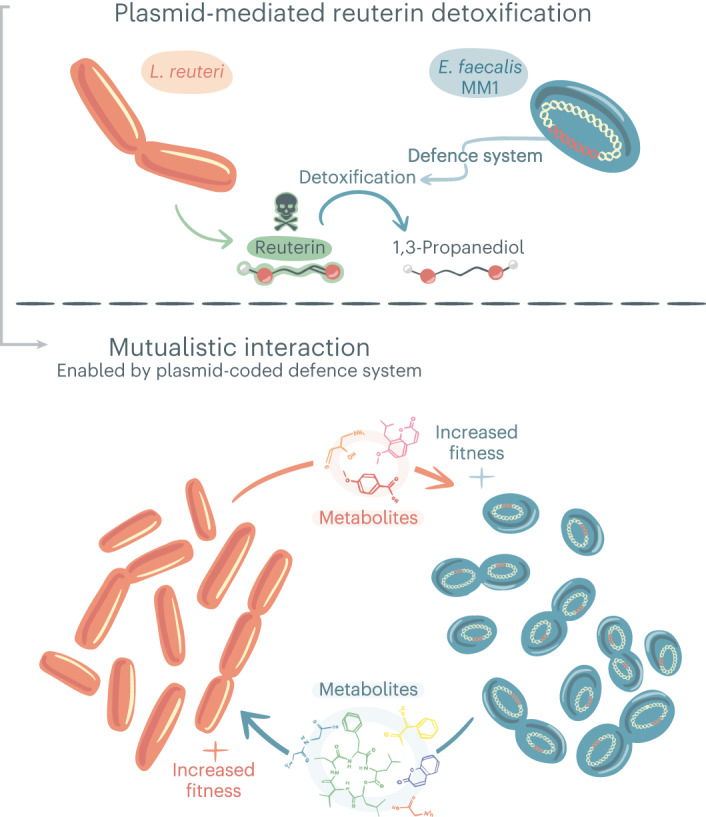
Schematic model of the findings revealed in our study. Our data demonstrate that the antibacterial toxin, reuterin, produced by *L. reuteri* (in orange) is detoxified by the plasmid-encoded defence gene (in red) of *E. faecalis* (in blue), thus allowing *E. faecalis* survival and use of *L. reuteri*-produced metabolites to increase its fitness. The beneficial effect becomes mutual when the metabolites produced by *E. faecalis* are consumed by *L. reuteri*, which in turn also increases the latter’s fitness.

**Extended Data Fig. 7:**
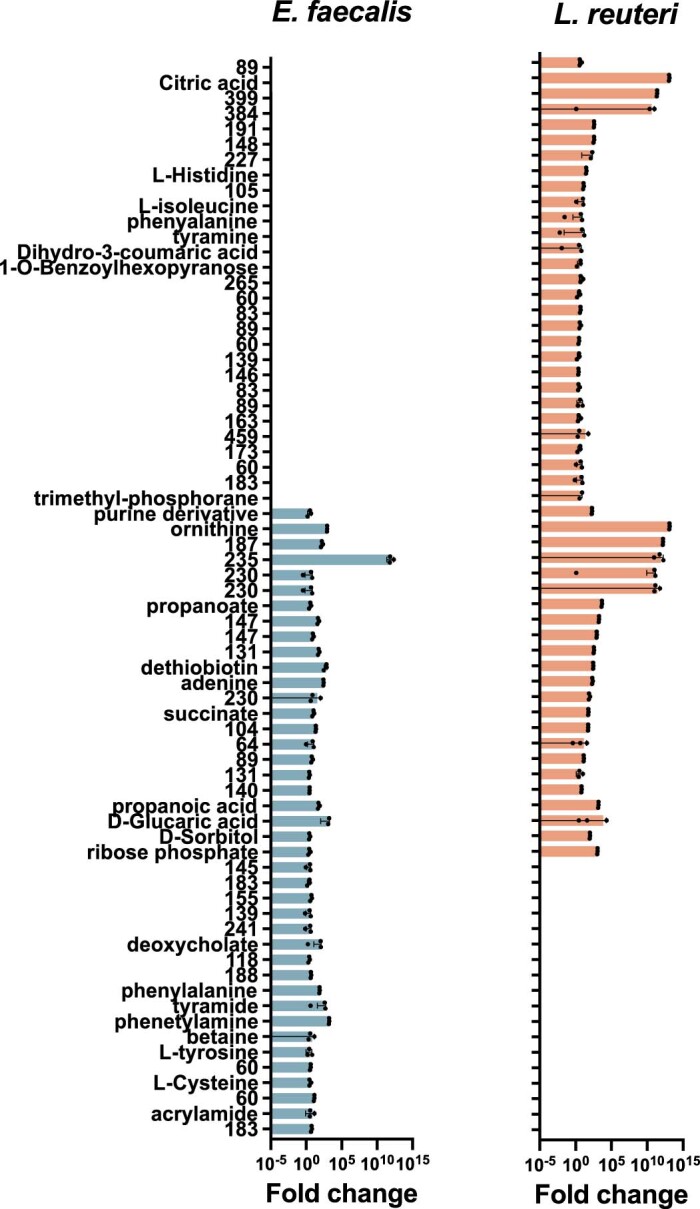
Production of metabolites by *L. reuteri* and *E. faecalis*. LC-MS analysis of the metabolites produced by *L. reuteri* and *E. faecalis* cultured on basal medium (as compared to a blank medium) and spent medium of each other (as compared to the spent medium of the first inoculated strain), as represented by graphs displaying the fold change of the produced metabolites (fold change for production defined as the ratio of [metabolites after growth/metabolites before growth] equal or superior to 2.5). All LC-MS measurements were performed on biological triplicates for which actual values, mean, and standard deviations are presented. The y-axis gives the mass of the metabolites (m/z) or the annotations when available. Source data

**Extended Data Fig. 8:**
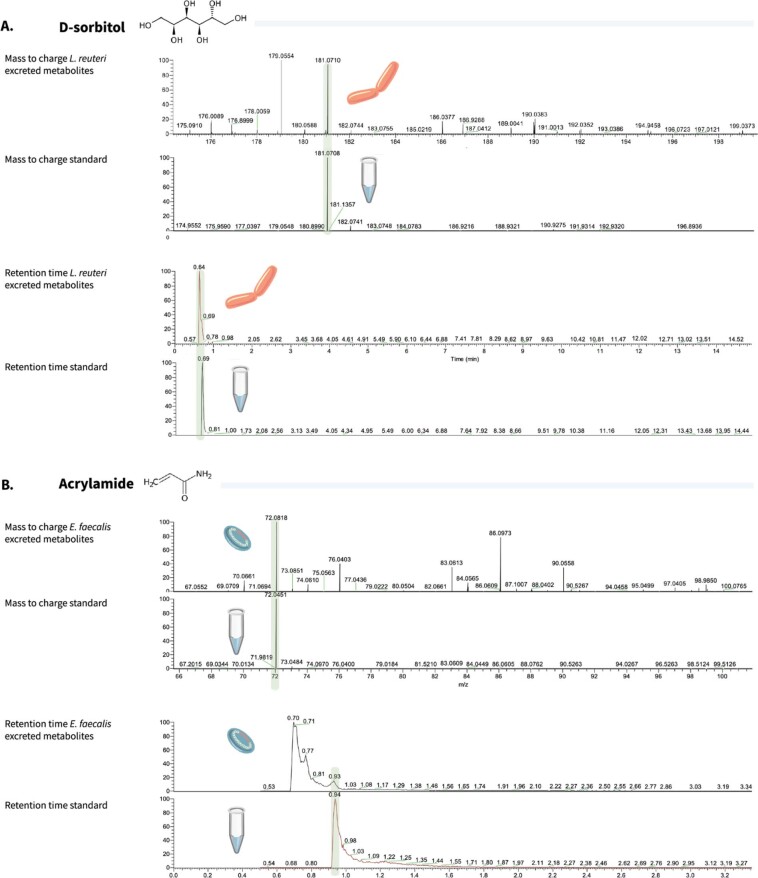
Validation of exchange metabolites by LC-MS using commercially available standards. Mass-to-charge and retention time chromatograms are given for D-sorbitol (A) and acrylamide (B) in experimental samples and standard solutions.

In view of the above, we performed an untargeted metabolomic analysis of production–consumption of metabolites of *E. faecalis* MM1 on spent medium of *L. reuteri* and vice versa (Supplementary Table 2). We detected a clear niche-partitioning pattern between the two microbes with regards to metabolite consumption, except for one metabolite consumed by both, suggesting that the two species are not competing for resources (Fig. 4b). Both microbes produce similar numbers of metabolites (Extended Data Fig. 7) and interestingly, the *E. faecalis* MM1 strain was able to consume 3 of the 52 metabolites identified to be produced by *L. reuteri*. Two of them were not present in the medium beforehand and were fully consumed by *E. faecalis* MM1. These metabolites could therefore be growth limiting and could potentially explain the faster growth of *E. faecalis* MM1 during the logarithmic stage on the spent media until they became limiting due to their total consumption and depletion by *E. faecalis* MM1. It should be noted that in the co-culture of both microbes, *E. faecalis* MM1 produces more biomass compared with its monoculture, potentially due to the higher availability of these metabolites under those conditions. Among the 41 metabolites produced by *E. faecalis* MM1, *L. reuteri* consumed 5, which were not fully consumed, thus explaining the higher biomass of this microbe grown on the spent medium, which corresponds to its growth in co-culture (Fig. 4a(iii)). In total, we annotated 29 metabolites out of the 84 metabolites of interest using five different platforms, some of which were reported in previous studies^38–42^ of *L. reuteri* and *E. faecalis*. The metabolites produced by *L. reuteri* and consumed by *E. faecalis* were annotated as d-sorbitol, d-glucaric acid and a purine metabolism derivative. Four out of the five metabolites produced by *E. faecalis* and consumed by *L. reuteri* were annotated as l-cysteine, ribose phosphate, acrylamide and betaine (Fig. 4c(i)). These annotations suggest that the two bacteria exchange metabolites that are not clustered in a specific pathway but spread across various pathways of their metabolism. We confirmed the annotations of acrylamide and d-sorbitol through LC–MS analysis using commercially available sources of these metabolites (Extended Data Fig. 8). Furthermore, we conducted growth experiments with *L. reuteri* and *E. faecalis* on acrylamide and d-sorbitol as the sole carbon sources, respectively, to confirm their utilization. Our results demonstrated that both bacterial strains were able to effectively utilize these compounds, resulting in positive effects on their growth (Fig. 4c(ii)). Altogether, our results suggest that metabolic exchange across the metabolic range between the two species is enabled by the maintenance of the defence plasmid coding for the 1,3-PD defence gene when reuterin is present in the environment (Fig. 5).

**Extended Data Fig. 9:**
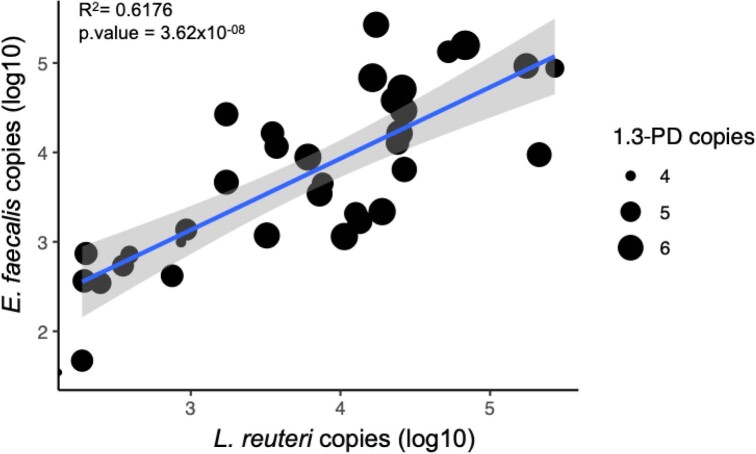
Linear regression model between *L. reuteri* and *E. faecalis* abundance in 34 rumen samples. The data was log10 transformed before fitting the linear model with the lm function in R (version 4.2.1). The simple linear regression model statistical test was used, and the *P* value two-sided with no correction for multiple comparisons. The shaded area in gray represents confidence intervals (95%). The circle sizes represent the 1,3-PD gene copies number. Source data

The observed beneficial interaction between *L. reuteri* and *E. faecalis* in vitro led us to investigate their relationship in vivo. We examined their co-occurrence and the presence of the 1,3-PD plasmidic gene in the rumen of 34 cows. Quantitative PCR analysis revealed a Spearman correlation of 0.82 between the two species (Fig. 4d(i)), and both *L. reuteri* and *E. faecalis* quantities were found to correlate with 1,3-PD plasmidic gene quantification, with Spearman correlations of 0.53 and 0.54, respectively (Fig. 4d(i)); fitting a linear regression model resulted in an *R*^2^ of 0.6176 (Extended Data Fig. 9), indicating that the observed beneficial interaction between these two species in vitro also occurs in natural environments (Fig. 4d(ii)). Moreover, an analysis of human gut plasmidomes from 311 faecal samples of healthy individuals revealed that 17% of the samples contained a plasmid with a putative 1,3-PD gene. This gene shared 44% amino-acid identity and 61% similarity to the rumen plasmid-borne 1,3-PD protein sequence. In addition, the putative 1,3-PD plasmid-borne gene was found to co-occur with *L. reuteri* (Fisher exact test, *P* < 1 × 10^−5^), indicating the widespread presence of this defence mechanism in gut environments where *L. reuteri* is present (Supplementary Table 3). These findings suggest that the observed in vitro interaction may be relevant in other gut environments as well.

## Discussion

In dense microbial communities, such as gut ecosystems and specifically in rumen ecosystems, microbial weapons and defence mechanisms play an instrumental role in the establishment and maintenance of microbial interactions. Such interactions are mostly established by competing for resources such as space and nutrients. Most of the research on bacterial warfare has focused on the benefit or cost from the direct outcome of using the weapon or the defence system on the microbial population or on the type of interaction, for example, surviving the attack or winning the competition. Plasmids are thought to be instrumental in the dispersal of such systems and their effect on microbial communities. Nevertheless, there are only a few (if any) examples of indirect effects of such mechanisms for establishing or changing the type of interaction between the warring parties within the community.

Here we highlight a unique, higher-order beneficial interaction type that is dependent on and mediated by the implementation of a defence mechanism that is coded on a plasmid. This plasmid accessory function confers upon its bacterial host the ability to maintain a beneficial relationship with an attacking microbial species. The aim of our work was to gain deep insight into how plasmids containing accessory genes can influence the ecology within their ecosystem. Focusing on the rumen ecosystem, we identified a plasmid carrying the 1,3-PD accessory gene that has high occupancy across ecosystems, and we performed an integrated top-down/bottom-up study involving bioinformatics, biochemical, molecular and cellular analyses. We found that carrying the plasmid provides the means to resist the toxic effect of reuterin in vitro and enabled *E. faecalis* to maintain a mutualistic metabolic interaction with *L. reuteri*, a toxin producer, thereby contributing to the fitness of both parties. Both the reuterin producer and the reuterin degrader were able to consume the produced metabolites of its counterpart, which suggests reciprocity among these cross-feeding bacterial species. Conferring an advantage that is specific only for the bacterial host of the plasmid would also account for the absence of mobility genes on the plasmid, thereby providing exclusivity for the beneficial effect of the plasmid-borne defence system.

In addition to these ecological implications, it seems that the high prevalence of the reuterin defence system on a plasmid would designate reuterin as an antibacterial toxin that has noticeable ecological significance and relevance in the rumen ecosystem, as well as gut ecosystems in other animal species. We find circumstantial evidence to support this notion as we observed the occurrence of this plasmidic gene in natural rumen samples with a positive correlation to both *E. faecalis* and *L. reuteri*, suggesting a potential mutually beneficial interaction in vivo. Furthermore, we found a homologous plasmidic 1,3-PD gene to be widespread in human gut environments where *L. reuteri* is present, indicating the potential for such interactions to occur in human gut microbiota. Nevertheless, such correlations should be further investigated to confirm in vivo interactions between these species. Hence future studies are needed to strengthen our primary findings and to decipher the importance of such interactions in vivo while focusing on the spatial organization of the interacting partners. The evolution of the toxin and defence gene in *L. reuteri* and *E. faecalis* is probably not driven by its role in enabling the interaction between these species. The production of reuterin has probably evolved primarily as a competitive mechanism, given its broad-spectrum toxin properties. The acquisition of the plasmid by *E. faecalis* may have occurred coincidentally along with its carried defence activity. Regardless of the chronological order of events, the presence of the defence system allows these two niche-partitioning non-competitive species to co-exist and support one another while excluding competitors.

To summarize, our findings show that a plasmid-coded trait has an important ecological role that is instrumental in bacterial warfare and interactions. They highlight the potential role of plasmids in facilitating mutualistic interactions between microbial species and emphasize plasmids’ importance as drivers of community dynamics and interactions. Our results surface a conceptual paradigm in microbial war and peace, perhaps analogous to an arms race in human societies, whereby possession of a defence system by one partner against the weapons system of another would enable a status quo of peace and prosperity.

## Methods

### Bioinformatic analysis of 1,3-PD-associated plasmid

The plasmidomes of 78 Holstein dairy cows were retrieved from the raw reads of sequenced metagenomes^16^ using the Recycler algorithm^17^. Plasmid containing 1,3-PD genes was retrieved using local Blast^43^. The plasmid was analysed for the presence of conjugative systems and relaxases using ConjScan^44^ of McSyFinder and MOBscan^45^. The raw reads of 311 healthy human individual faecal samples from project SRP100518 were trimmed using Trim Galore^46^, assembled into contigs by Megahit^47^ and plasmids were recovered by SCAPP^48^. Next, the amino-acid sequence of the 1,3-PD gene from the rumen plasmidome was blasted against these plasmids, resulting in the retrieval of a plasmid encoding a putative 1,3-PD gene with 44% amino-acid identity and 61% similarity to the rumen plasmidome 1,3-PD protein sequence. Finally, raw reads of each of the 311 samples were mapped to this plasmid using BBmap^49^, and the plasmid was considered present in a sample if its read coverage was higher than 70% as computed by SAMtools mpileup^50^.

### 1,3-PD gene cloning and protein purification

The gene coding for 1,3-PD was synthesized and cloned into plasmid pSH21 such that the expressed protein presents an N-terminal polyhistidine tag (Supplementary Table 4). The resulting plasmid was inserted to *E. coli* ER2566 competent cells and the gene was expressed under the transcriptional control of the T7 promoter in the presence of 100 µg ml^−1^ ampicillin. The protein was initially purified using Ni-NTA beads, followed by size exclusion chromatography using a GE Healthcare 16/60 Superdex 75 column that was equilibrated with 50 mM HEPES pH 7.5 and 100 mM NaCl. The purified protein was stored without glycerol at −80 °C.

### Enzymatic activity of 1,3-PD

The putative enzyme performs the reaction 1,3-PD + NAD^+^->NADH + reuterin and vice versa. Since purified reuterin is not commercially available, the enzymatic activity was measured using 1,3-propanediol and NAD^+^ as substrates. 1,3-Propanediol at 2 mM was used as a substrate in the presence of 1 mM NAD^+^ to measure the activity of the enzyme (1 µM). The absorbance at 340 nm was used to measure the production of NADH. The reaction was performed for 15 min at 37 °C. Kinetic parameters were determined via titration experiments by fitting a Michaelis–Menten model to the data.

### Reuterin production by *L. reuteri*

The reuterin producer, *L. reuteri*, was used to produce reuterin from glycerol, as it converts glycerol to reuterin in the presence of glucose^24^. *L. reuteri* strain ATCC 6475 was grown on MRS medium (Difco) under anaerobic conditions for 24 h at 37 °C. The bacterial culture was then centrifuged under anaerobic conditions for 3 min at 5,000*g*. The supernatant was discarded, the cells were washed quickly with saline solution and resuspended in 1 ml of 250 mM glycerol, supplemented with 0.1% glucose and incubated at 37 °C for 2 h under anaerobic conditions. Then, after a centrifugation step at 12,000 *g* for 5 min, the aqueous supernatant containing the produced reuterin was filtered and kept at 4 °C. Aliquots of 50 μl of the supernatant or acrolein (Sigma-Aldrich) at concentrations ranging from 0 to 70 nM, or double-distilled water (negative control), were added to 37.5 μl tryptophan (0.01 M solution in 0.05 M HCl), together with 150 μl of concentrated HCl in 1.5 ml tubes, and the solution was incubated for 30 min at 37 °C. Absorbance, measured at 560 nm, was used to calculate the concentrations of reuterin produced by *L. reuteri*.

### 1,3-PD-based protection against reuterin

The gene coding for 1,3-PD was cloned into pBR322 using specific primers (Supplementary Table 4) using the Gibson assembly technique to form pBR322:1,3-PD. The plasmid was then transformed to *E. coli* ER2566 cells in the presence of 100 μg ml^−1^ ampicillin. The plasmid pBR322 allows constitutive expression of the 1,3-PD gene. The wild-type strain ER2566 containing pBR322 alone and the ER2566 strain containing pBR322:1,3-PD were grown in 96-well plates with increasing concentrations of reuterin (0 to 1 mM) in 200 μl and incubated for 24 h at 37 °C (volumes of cultures were homogenized across samples using sterile water). Measurements of optical density (OD)_600nm_ after the incubation period were used to determine the protection against the reuterin compound by 1,3-PD. Similarly, *E. faecalis* MM1, DSM 8630 pMM1 and DSM 8630 ∅ were grown in increasing concentrations of reuterin (0 to 0.9 mM solution) in 1,000 μl of basal defined medium (volumes of cultures were homogenized across samples using sterile water), incubated at 37 °C for 24 h and measured for OD_600_.

### Preparation of reuterin standard

Three ml of reuterin produced by *L. reuteri* as described above was taken to prepare the reuterin standard using purification on a silica gel column as previously described^51^. The elution was monitored by adding a droplet of the eluent on a silica gel 60 thin-layer chromatography plate (Merck), which was immediately dipped into a 2% (w/v) Purpald reagent solution (Aldrich) in 1 M NaOH. Fractions with reuterin were combined and the solvent was evaporated under reduced pressure. The dry residue was weighed and immediately redissolved in double-distilled water to 2 mg ml^−1^. The obtained solution was stored at −20 °C until preparation of the standard curve.

### Measurement of reuterin

A number of 20 rumen samples taken from a previously published cow cohort^16^ were analysed for reuterin concentration using LC–MS. Reuterin was derivatized as previously described^52^ and analysed using an LC–MS/MS instrument consisting of an Acquity I-class UPLC system (Waters) and Xevo TQ-S triple quadrupole mass spectrometer (Waters) equipped with an electrospray ion source. MassLynx and TargetLynx software (v.4.2, Waters) were applied for the acquisition and analysis of data. Chromatographic separation was performed on a 50 mm × 2.1-mm-internal-diameter, 1.7-μm UPLC BEH C18 column (Waters Acquity), with 0.1% formic acid as mobile phase A and methanol as mobile phase B at a flow rate of 0.3 ml min^−1^, and column temperature of 35 °C. A gradient was used as follows: a slight linear increase of B from 0.1 to 0.2% for 0–1.5 min; then a linear increase to 50% B for 5 min, held at 50% B for 0.5 min, back to 0.1% B within 0.5 min and equilibration at 0.1% B for 2 min. Injection volume was 3 μl.

### Enrichment of reuterin-resistant bacteria

A cow sample in which the plasmid was detected was grown on M10 medium containing volatile fatty acids and supplemented with 5 g l^−1^ fructose in liquid media and cultured via passaging for 7 d to reach typical richness reduction resulting from growth in tubes. The bacterial community was further grown in the same medium (in 1 ml volumes, supplemented with 25 μl reuterin (0.25 mM final concentration, which approximately corresponds to the average reuterin concentration detected in samples in which reuterin was present) or 25 μl double-distilled water) and cultured by passaging for 7 d. The final cultures were observed under a light microscope and examined using in-house 16S rRNA gene-amplicon Illumina sequencing as previously described^53^. An exact sequence variants table was created using DADA2 and QIIME^54,55^.

### Isolation of plasmid-containing bacteria

The supernatant fluids of the enriched culture above (containing various metabolites excreted by the community) were filtered using a 20-μm-pore filter and used to enrich M10 agar plates containing volatile fatty acids and supplemented with 5 g l^−1^ fructose. In addition, 0.1 mM reuterin was added to the plates to enrich for plasmid-containing bacteria. The obtained colonies were grown individually and subjected to miniprep preparation (RBC miniprep kit). Using specific primers for the 1,3-PD gene (Supplementary Table 1), PCR was performed on the purified plasmids using Phusion polymerase (NEB). Positive colonies were sent for 16S Sanger sequencing. To ensure the circular nature of the PCR product, all-around PCR was performed using the reverse complements of the specific 1,3-PD primers. The plasmid was then sequenced by Sanger sequencing and gene walking to fully validate the plasmid sequence.

### Genomic comparison

The genomic DNA of *E. faecalis* DSM 8630 and MM1 strains was extracted using the phenol–chloroform method^56^ and the DNA was sequenced using an Illumina sequencing NovaSeq S1. The sequences were assembled with SPAdes^57^ and deposited in GenBank (accessions numbers JAMKBU000000000 and JAMKBV000000000).

The DNA contents of the two strains were compared using Mauve^58^, and Proteinortho^59^ was used to compare orthologous groups from both strains. A heat map of the orthologous group was created using Superheat in R (https://rlbarter.github.io/superheat/).

### CARD-FISH

Long probes (~300 bases) were designed for 1,3-PD and GroEL genes to perform standard CARD-FISH (Supplementary Table 4). PCR was performed using Ex *Taq* polymerase (TaKaRa) following manufacturer protocol and replacing 90% of the dTTP volume with Dig-11-dUTP (Merck).

Plasmids were stained using DNA CARD-FISH. All centrifugations were carried out at 5,000 *g* at room temperature for 5 min. *E. faecalis* MM1 or DSM 8630 strains were grown overnight in brain-heart infusion medium (Difco) at 37 °C and the pellets of 1 ml cultures were fixed with 4% paraformaldehyde for 15 min. Fixed cells were then centrifuged and permeabilized with lysis buffer containing 0.05 M EDTA pH 8, 0.1 M Tris-HCl pH 8 and lysozyme (Merck) at 3 mg ml^−1^ in PBS. Lysis occurred at 4 °C for 15 min. Cells were then centrifuged before being resuspended in hybridization buffer (20% dextran sulfate, 20 mM Tris-HCl, 0.1% SDS, 0.8 M NaCl, 1% blocking reagent and 35% formamide). The reaction was incubated at 42 °C for 30 min and 5 μM of the probe was added to the reaction. Cells were then placed at 95 °C for 10 min, incubated at 42 °C overnight and washed twice in hybridization buffer lacking formamide and dextran sulfate. The cells were then resuspended in antibody binding solution (1% Western blocking reagent in PBS, supplemented with 0.1% Pluronic F-68 reagent) and incubated for 45 min with rotation to block non-specific binding. Subsequently, 2 μl of anti-digoxigenin-POD Fab fragments (Roche) was added and the cells were incubated for another 1.5 h. Cells were washed twice in PBS. The cell pellet was then resuspended in 100 μl amplification solution (5 M NaCl, 20% dextran sulfate and 0.05% Western blocking reagent solution in PBS). Then, 1 μl 0.15% H_2_O_2_ and 0.1 μl Alexa Fluor 647 Tyramide reagent (ThermoFisher) were added to the mixture, the cells were incubated at 37 °C for 45 min in the dark, following which, 1 ml PBS was added and the cells were centrifuged. Finally, the cells were washed twice in PBS and then counterstained with DAPI (1 µg ml^−1^). Images were acquired using a 3i Marianas spinning disk confocal microscope equipped with a Yokogawa W1 module and Prime 95B sCMOS camera. Excitation laser (absorbance 637 nm and emission filter 672–712 nm) was achieved using a ×100 Zeiss Plan-Apochromat 1.4 NA DIC oil objective.

### STORM

Stained *E. faecalis* MM1 cells (20 μl) were immobilized on poly-l-lysine-treated STORM high glass-bottom 35-mm μ-Dish (ibidi, 81158) and imaged by direct STORM. Photoswitching of the Alexa 647 dye was induced by a STORM buffer: 7 μM glucose oxidase (Sigma), 56 nM catalase (Sigma), 5 mM cysteamine (Sigma), 50 mM Tris, 10 mM NaCl and 10% glucose (pH 8)^60^. Image acquisition and data reconstruction were performed using the Elyra PS1 system (×100 oil objective) and Zen software (Zeiss).

### Genetic manipulation

Full-length plasmid from *E. faecalis* MM1 was cloned into pAM401 (ref. ^61^) into XbaI and Sal restriction sites and transformed into *E. faecalis* DSM 8630 competent cells by electroporation^62^, generating *E. faecalis* DSM 8630 pMM1. In parallel, pAM401 only was also transformed into *E. faecalis* DSM 8630 competent cells to serve as a negative control (*E. faecalis* DSM 8630 ∅).

### Growth curves

*E. faecalis* MM1 or *L. reuteri* was grown at 37 °C in 0.2 ml basal defined medium for 20 h in 96-well plates in an OD plate reader (800TS absorbance reader, Biotek) under anaerobic conditions. Basal defined medium was prepared as described in Supplementary Table 5. All chemicals were ordered from Sigma-Aldrich, except for the amino acids (FORMEDIUM).

Mono- and co-cultures were performed by inoculating 100 μl of fresh cultures in the same media in 8 ml volumes in Hungate tubes with addition of 0.25 mM reuterin, and incubating for 20 h at 37 °C. Given that *L. reuteri* produces reuterin during the late stationary phase, by including reuterin throughout the experiment, we aimed to mimic the conditions that more closely resemble the natural environment and the prevailing concentrations of reuterin in the rumen.

Supplementation assays were performed by adding 0.2 g l^−1^ of acrylamide or sorbitol to basal defined medium without glucose before growing *L. reuteri* or *E. faecalis*, respectively, for 20 h at 37 °C. Acrylamide supplementation assays were performed in Hungate tubes to avoid acrylamide evaporation.

### Quantitative PCR

Quantitative PCR was used to determine the copy number of the *E. faecalis* strains and *L. reuteri* in single and co-cultures. The pellets of 1 ml cultures were obtained by centrifugation at 4,000 *g* for 5 min and the genomic DNA was extracted as performed in ref. ^56^ without the initial filtration steps. A volume of 2 μl of a 10-fold dilution was used in the reaction mix described below.

Standard curves suitable for the quantification of *Enterococcus-*specific and *L. reuteri* 16S rRNA genes were generated by amplifying serial 10-fold dilutions of quantified gel-extracted PCR products, obtained by amplification of each fragment (primers in Supplementary Table 4). The standard curves were obtained using 8 dilution points and were calculated using the Rotorgene 6000 series software (Qiagen). Real-time PCR was performed in a 10-μl reaction mixture containing 5 μl Absolute blue SYBR green master mix (Thermo Scientific), 0.5 μl of each primer (at 10 mM) and 2 μl of the DNA template.

Quantitative PCR was also used to determine the copy number of *E. faecalis*, *L. reuteri* and 1,3-PD genes in metagenomic DNA extracted from a cohort of 34 cows^16^. Copy numbers of each gene were normalized to the DNA concentration of each sample measured using a Qubit dsDNA High Sensitivity Assay kit (Invitrogen). The obtained plasmid quantities confirmed Recycler predictions and increased the detection level in additional samples. Linear regression was used to measure the relationship between the absolute abundance of *L. reuteri* and *E. faecalis* in these samples. The data were log_10_ transformed before fitting the linear model with the lm function in R (v.4.2.1).

### Proximity assay

Proximity assay was performed on 1-well plates containing basal defined medium supplemented with 1.5% agar with 0.25 mM reuterin. Plates were pre-incubated at room temperature for 24 h to avoid bacterial smears. *E. faecalis* and *L. reuteri* strains were spotted on the plates by depositing a volume of 2.5 μl each at 1-cm distance and incubated at 37 °C for 48 h under anaerobic conditions. The assay was performed with either *E. faecalis* MM1, DSM 8630 pMM1 or DSM 8630 ∅.

After incubation, the distance between *L. reuteri* and *E. faecalis* colonies was measured, as well as the diameter of each colony.

### Untargeted LC–MS analysis of metabolomes

*E. faecalis* MM1 was grown on basal defined medium alone or mixed with equal volumes of the spent medium of *L. reuteri* ATCC 6475 (0.2 ml total volume distributed in 96-well plates). Similarly, *L. reuteri* ATCC 6475 was grown for 24 h at 37 °C on basal defined medium alone or mixed with equal volumes of the spent medium of *E. faecalis* MM1. Supernatant fluids of all cultures were filter-sterilized for subsequent LC–MS analysis.

For metabolite extraction, a volume of 100 μl from each supernatant fraction was mixed with 1 ml of pre-cooled metabolomics extraction mixture (methanol containing 2.5 μg ml^−1^ ampicillin). Tubes were incubated for 10 min in an orbital shaker before centrifugation at full speed for 10 min.

For polar metabolite analysis, 150 μl of extracted metabolites or standards in LC–MS grade water was transferred to glass vials and kept at −20 °C until used. A volume of 5 μl of each sample was injected into a C18 100 mm × 2.1 mm × 1.7 μm reversed-phase column (Waters) using a Waters Acquity UPLC system. Mobile phases consisted of 0.1% formic acid (A), and acetonitrile supplemented with 0.1% formic acid (B) (UPLC MS grade, BioSolve). The metabolites were eluted at a flow rate of 400 μl min^−1^, using the following percentages of A: 1-min gradient from 99 to 95%; 7-min gradient from 95 to 1%; 2.9 min at 1%; 0.1-min gradient from 1 to 5%; 2-min gradient from 5 to 99%, and 2 min at 99% for washing and re-equilibration of the column (15 min total run time). The mass spectra were acquired using an Exactive mass spectrometer (ThermoFisher). The spectra were recorded using both full scan and all-ion fragmentation scan mode, covering a mass range of 100–1,500 *m*/*z*. The resolution was set to 70,000 with 10 scans per second, restricting the Orbitrap loading time to a maximum of 100 ms with a target value of 1 × 10^5^ ions. The capillary voltage was set to 3.5 kV, with a sheath gas flow value of 60 and an auxiliary gas flow of 20. The capillary temperature was set at 275 °C, while the drying gas in the heated electrospray source was set at 300 °C. Chromatogram processing, peak detection and integration were performed using MZmine 2, with signal to noise ratio set to 5 (MZmine 2.41.2)^63^. Production of metabolites was defined as a ratio of ‘metabolites after growth/metabolites before growth’ equal or superior to 2.5. Consumption of metabolites was defined as the same ratio equal or inferior to 0.4. To increase stringency, production or consumption of a metabolite was further confirmed if the metabolite was produced or consumed in three biological replicates. Metabolites that were neither produced nor consumed by *E. faecalis* or *L. reuteri* were ignored. Metabolite annotations were performed by matching masses and retention times obtained by running the commercial Mass Spectrometry Metabolite Library of Standards (MSMLS, Iroa Technologies). Additional annotations were obtained by: performing Molecular Networking using GNPS^64^, using the Compound Discoverer v.3.3 (Thermo Scientific), extracting the metabolites present in the KEGG genomes of the bacteria^40,65^ and using MetaboAnalyst^66^. The latter tool was used to detect peaks that corresponded to the same compound with various ionizations. For these metabolites, we chose the ionized compound that was better detected as a representative peak.

Commercial acrylamide and d-sorbitol were purchased (Merck) to validate their annotations, using the workflow described above.

### Statistics and reproducibility

No statistical method was used to predetermine sample size. No data were excluded from the analyses. The experiments were not randomized. Data distribution was assumed to be normal, but this was not formally tested (individual datapoints are presented in the figures). The Investigators were not blinded to allocation during experiments and outcome assessment.

### Reporting summary

Further information on research design is available in the Nature Portfolio Reporting Summary linked to this article.

### Supplementary information

Reporting Summary

Supplementary Tables Supplementary Table 1. Nucleotide sequence of *E. faecalis* MM1 plasmid and plasmid identified in the human gut-associated plasmidome. Table 2. Metabolomic raw data. Table 3. Analysis of human gut plasmidomes from 311 faecal samples of healthy individuals revealed that 17% of the samples contained a plasmid with a putative 1,3-PD gene. The putative 1,3-PD plasmid-borne gene co-occurs with *L. reuteri* (Fisher exact test, *P* < 1 × 10^−5^). Table 4. Primers used in the study. Table 5. Basal defined medium preparation.

### Source data

Source Data Fig. 1 Newick tree.

Source Data Fig. 2 Source data.

Source Data Fig. 3 Source data.

Source Data Fig. 4 Source data.

Source Data Extended Data Fig. 2 Source data.

Source Data Extended Data Fig. 3 Source data.

Source Data Extended Data Fig. 4 Source image.

Source Data Extended Data Fig. 6 Source data.

Source Data Extended Data Fig. 7 Source data.

Source Data Extended Data Fig. 9 Source data.

## Figures and Tables

**Extended Data Fig. Fig. 1:**
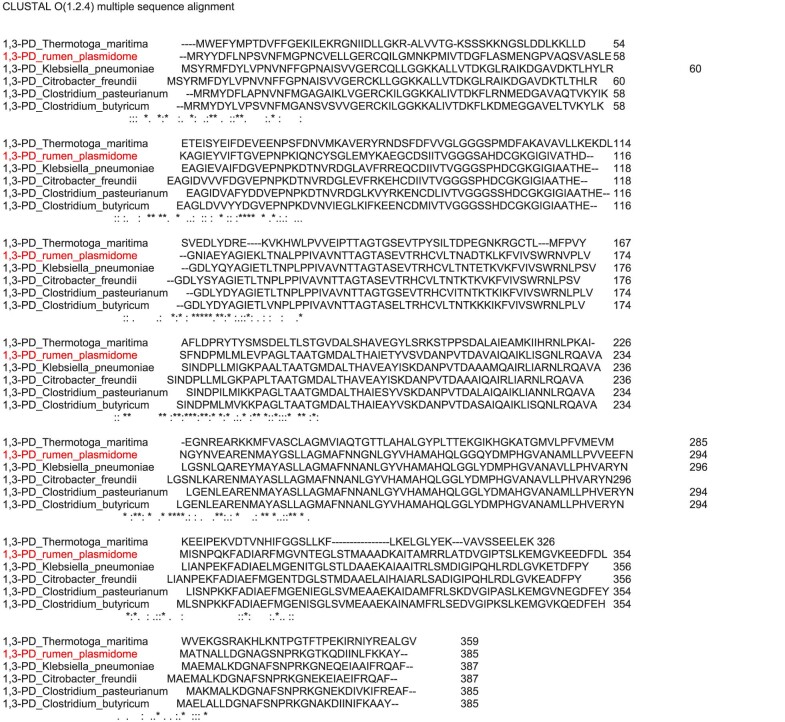
Multiple sequence alignment of 1,3-PD genes. Amino-acid sequence alignment of the putative rumen plasmid 1,3-PD gene variant shows high similarities with other previously characterized enzymes from this family.

## Data Availability

Genome sequencing and 16S rRNA sequences of *E. faecalis* MM1 and DSM 8630 have been deposited in GenBank (JAMKBV000000000, JAMKBU000000000, OR513053 and OR513080). Metabolomics data are deposited in MetaboLights^67^ under the identifier MTBLS7248 (https://www.ebi.ac.uk/metabolights/editor/MTBLS7248/descriptors). Source data are provided with this paper.

## References

[1] Kado CI (1998). Origin and evolution of plasmids. Antonie Van Leeuwenhoek.

[2] Heuer H, Abdo Z, Smalla K (2008). Patchy distribution of flexible genetic elements in bacterial populations mediates robustness to environmental uncertainty. FEMS Microbiol. Ecol..

[3] Nuti, M. P., Lepidi, A. A., Prakash, R. K., Hooykaas, P. J. J. & Schilperoort, R. A. in *Molecular Biology of Plant Tumors* (eds Kahl, G. & Schell, J. S.) 561–588 (Academic Press, 1982).

[4] Zwanzig M (2021). The ecology of plasmid-coded antibiotic resistance: a basic framework for experimental research and modeling. Comput. Struct. Biotechnol. J..

[5] Frost LS, Leplae R, Summers AO, Toussaint A (2005). Mobile genetic elements: the agents of open source evolution. Nat. Rev. Microbiol..

[6] Novick RP (2003). Mobile genetic elements and bacterial toxinoses: the superantigen-encoding pathogenicity islands of *Staphylococcus aureus*. Plasmid.

[7] Crossman LC (2005). Plasmid replicons of *Rhizobium*. Biochem. Soc. Trans..

[8] van der Meer JR, Sentchilo V (2003). Genomic islands and the evolution of catabolic pathways in bacteria. Curr. Opin. Biotechnol..

[9] Gil R, Sabater-Muñoz B, Perez-Brocal V, Silva FJ, Latorre A (2006). Plasmids in the aphid endosymbiont *Buchnera aphidicola* with the smallest genomes. A puzzling evolutionary story. Gene.

[10] Shterzer N, Mizrahi I (2015). The animal gut as a melting pot for horizontal gene transfer. Can. J. Microbiol..

[11] Granato ET, Meiller-Legrand TA, Foster KR (2019). The evolution and ecology of bacterial warfare. Curr. Biol..

[12] Morais S, Mizrahi I (2019). Islands in the stream: from individual to communal fiber degradation in the rumen ecosystem. FEMS Rev. Microbiol..

[13] Mizrahi I, Wallace RJ, Moraïs S (2021). The rumen microbiome: balancing food security and environmental impacts. Nat. Rev. Microbiol..

[14] Kav AB (2012). Insights into the bovine rumen plasmidome. Proc. Natl Acad. Sci. USA.

[15] Brown Kav A (2020). Unravelling plasmidome distribution and interaction with its hosting microbiome. Environ. Microbiol..

[16] Shabat SKB (2016). Specific microbiome-dependent mechanisms underlie the energy harvest efficiency of ruminants. ISME J..

[17] Rozov R (2017). Recycler: an algorithm for detecting plasmids from de novo assembly graphs. Bioinformatics.

[18] Shapiro JT (2023). Multilayer networks of plasmid genetic similarity reveal potential pathways of gene transmission. ISME J..

[19] Brown Kav A, Benhar I, Mizrahi I (2013). A method for purifying high quality and high yield plasmid DNA for metagenomic and deep sequencing approaches. J. Microbiol. Methods.

[20] Humphrey S (2021). Staphylococcal phages and pathogenicity islands drive plasmid evolution. Nat. Commun..

[21] Frickey T, Lupas AN (2004). PhyloGenie: automated phylome generation and analysis. Nucleic Acids Res..

[22] Raynaud C, Sarçabal P, Meynial-Salles I, Croux C, Soucaille P (2003). Molecular characterization of the 1,3-propanediol (1,3-PD) operon of *Clostridium butyricum*. Proc. Natl Acad. Sci. USA.

[23] Smillie C, Garcillán-Barcia MP, Francia MV, Rocha Eduardo PC, de la Cruz F (2010). Mobility of plasmids. Microbiol. Mol. Biol. Rev..

[24] Talarico TL, Casas IA, Chung TC, Dobrogosz WJ (1988). Production and isolation of reuterin, a growth inhibitor produced by *Lactobacillus reuteri*. Antimicrob. Agents Chemother..

[25] Degnan PH, Taga ME, Goodman AL (2014). Vitamin B12 as a modulator of gut microbial ecology. Cell Metab..

[26] Daniel R, Boenigk R, Gottschalk G (1995). Purification of 1,3-propanediol dehydrogenase from *Citrobacter freundii* and cloning, sequencing, and overexpression of the corresponding gene in *Escherichia coli*. J. Bacteriol..

[27] Marçal D, Rêgo AT, Carrondo MA, Enguita FJ (2009). 1,3-propanediol dehydrogenase from *Klebsiella pneumoniae*: decameric quaternary structure and possible subunit cooperativity. J. Bacteriol..

[28] Luers F, Seyfried M, Daniel R, Gottschalk G (1997). Glycerol conversion to 1,3-propanediol by *Clostridium pasteurianum*: cloning and expression of the gene encoding 1,3-propanediol dehydrogenase. FEMS Microbiol. Lett..

[29] Johnson EA, Lin EC (1987). *Klebsiella pneumoniae* 1,3-propanediol:NAD+ oxidoreductase. J. Bacteriol..

[30] Cleusix V, Lacroix C, Vollenweider S, Duboux M, Le Blay G (2007). Inhibitory activity spectrum of reuterin produced by *Lactobacillus reuteri* against intestinal bacteria. BMC Microbiol..

[31] Goris J (2007). DNA-DNA hybridization values and their relationship to whole-genome sequence similarities. Int. J. Syst. Evol. Microbiol..

[32] Fautin DG (1991). The anemonefish symbiosis: what is known and what is not. Symbiosis.

[33] Korem T (2015). Growth dynamics of gut microbiota in health and disease inferred from single metagenomic samples. Science.

[34] Castellani C (2021). Production, storage stability, and susceptibility testing of reuterin and its impact on the murine fecal microbiome and volatile organic compound profile. Front. Microbiol..

[35] Zhang J, Sturla S, Lacroix C, Schwab C (2018). Gut microbial glycerol metabolism as an endogenous acrolein source. mBio.

[36] Engels C (2016). Acrolein contributes strongly to antimicrobial and heterocyclic amine transformation activities of reuterin. Sci. Rep..

[37] Morita H (2008). Comparative genome analysis of *Lactobacillus reuteri* and *Lactobacillus fermentum* reveal a genomic island for reuterin and cobalamin production. DNA Res..

[38] Keogh D (2016). Enterococcal metabolite cues facilitate interspecies niche modulation and polymicrobial infection. Cell Host Microbe.

[39] Ryu HW, Wee YJ (2001). Characterization of bioconversion of fumarate to succinate by alginate immobilized *Enterococcus faecalis* RKY1. Appl. Biochem. Biotechnol..

[40] Pérez Escriva P, Fuhrer T, Sauer U (2022). Distinct N and C cross-feeding networks in a synthetic mouse gut consortium. mSystems.

[41] Kaneuchi C, Seki M, Komagata K (1988). Production of succinic acid from citric acid and related acids by *Lactobacillus* strains. Appl. Environ. Microbiol..

[42] Kristjansdottir T (2019). A metabolic reconstruction of *Lactobacillus reuteri* JCM 1112 and analysis of its potential as a cell factory. Microb. Cell Fact..

[43] Altschul SF (1997). Gapped BLAST and PSI-BLAST: a new generation of protein database search programs. Nucleic Acids Res..

[44] Cury J, Abby SS, Doppelt-Azeroual O, Néron B, Rocha EPC (2020). Identifying conjugative plasmids and integrative conjugative elements with CONJscan. Methods Mol. Biol..

[45] Garcillán-Barcia MP, Redondo-Salvo S, Vielva L, de la Cruz F (2020). MOBscan: automated annotation of MOB relaxases. Methods Mol. Biol..

[46] Krueger, F. et al. FelixKrueger/TrimGalore: v0.6.10 - add default decompression path (0.6.10). *Zenodo*10.5281/zenodo.7598955 (2023).

[47] Li D, Liu C-M, Luo R, Sadakane K, Lam T-W (2015). MEGAHIT: an ultra-fast single-node solution for large and complex metagenomics assembly via succinct de Bruijn graph. Bioinformatics.

[48] Pellow D (2021). SCAPP: an algorithm for improved plasmid assembly in metagenomes. Microbiome.

[49] Bushnell, B. *BBMap: A Fast, Accurate, Splice-aware Aligner. No. LBNL-7065E* (Ernest Orlando Lawrence Berkeley National Laboratory, 2014).

[50] Li H (2009). The Sequence Alignment/Map format and SAMtools. Bioinformatics.

[51] Vollenweider S, Grassi G, König I, Puhan Z (2003). Purification and structural characterization of 3-hydroxypropionaldehyde and its derivatives. J. Agric. Food Chem..

[52] Yasuo M (2019). The relationship between acrolein and oxidative stress in COPD: in systemic plasma and in local lung tissue. Int. J. Chron. Obstruct. Pulmon. Dis..

[53] Furman, O. et al. Stochasticity constrained by deterministic effects of diet and age drive rumen microbiome assembly dynamics. *Nat. Commun.* 11, 1904 (2020). 10.1038/s41467-020-15652-8 PMC7170844 32312972

[54] Caporaso JG (2010). QIIME allows analysis of high-throughput community sequencing data. Nat. Methods.

[55] Callahan BJ (2016). DADA2: high-resolution sample inference from Illumina amplicon data. Nat. Methods.

[56] Stevenson DM, Weimer PJ (2007). Dominance of *Prevotella* and low abundance of classical ruminal bacterial species in the bovine rumen revealed by relative quantification real-time PCR. Appl. Microbiol. Biotechnol..

[57] Bankevich A (2012). SPAdes: a new genome assembly algorithm and its applications to single-cell sequencing. J. Comput. Biol..

[58] Darling ACE, Mau B, Blattner FR, Perna NT (2004). Mauve: multiple alignment of conserved genomic sequence with rearrangements. Genome Res..

[59] Lechner M (2011). Proteinortho: detection of (co-)orthologs in large-scale analysis. BMC Bioinformatics.

[60] Goliand I (2018). Resolving ESCRT-III spirals at the intercellular bridge of dividing cells using 3D STORM. Cell Rep..

[61] Fujimoto S, Ike Y (2001). pAM401-based shuttle vectors that enable overexpression of promoterless genes and one-step purification of tag fusion proteins directly from *Enterococcus faecalis*. Appl. Environ. Microbiol..

[62] Holo H, Nes IF (1989). High-frequency transformation, by electroporation, of *Lactococcus lactis* subsp. *cremoris* grown with glycine in osmotically stabilized media. Appl. Environ. Microbiol..

[63] Pluskal T, Castillo S, Villar-Briones A, Oresic M (2010). MZmine 2: modular framework for processing, visualizing, and analyzing mass spectrometry-based molecular profile data. BMC Bioinformatics.

[64] Wang M (2016). Sharing and community curation of mass spectrometry data with Global Natural Products Social Molecular Networking. Nat. Biotechnol..

[65] Vieira-Silva S (2016). Species–function relationships shape ecological properties of the human gut microbiome. Nat. Microbiol..

[66] Pang Z (2022). Using MetaboAnalyst 5.0 for LC–HRMS spectra processing, multi-omics integration and covariate adjustment of global metabolomics data. Nat. Protoc..

[67] Haug K (2020). MetaboLights: a resource evolving in response to the needs of its scientific community. Nucleic Acids Res..

