# Supplementary material for: Plasmid-encoded toxin defence mediates mutualistic microbial interactions
Source: Nat Microbiol. 2023 Dec 27;9(1):108–19. doi: 10.1038/s41564-023-01521-9 (PMC10769881; doi:10.1038/s41564-023-01521-9)
Supplement: Supplementary file 1 — Reporting Summary [file 41564_2023_1521_MOESM1_ESM.pdf]

nature portfolio

Corresponding author(s): Itzhak Mizrahi

Last updated by author(s): Sep 1, 2023

Reporting Summary

Nature Portfolio wishes to improve the reproducibility of the work that we publish. This form provides structure for consistency and transparency in reporting. For further information on Nature Portfolio policies, see our Editorial Policies and the Editorial Policy Checklist.

Statistics

For all statistical analyses, confirm that the following items are present in the figure legend, table legend, main text, or Methods section.

n/a	Confirmed	
☐	☒	The exact sample size (*n*) for each experimental group/condition, given as a discrete number and unit of measurement
☒	☐	A statement on whether measurements were taken from distinct samples or whether the same sample was measured repeatedly
☐	☒	The statistical test(s) used AND whether they are one- or two-sided<br>*Only common tests should be described solely by name; describe more complex techniques in the Methods section.*
☒	☐	A description of all covariates tested
☒	☐	A description of any assumptions or corrections, such as tests of normality and adjustment for multiple comparisons
☐	☒	A full description of the statistical parameters including central tendency (e.g. means) or other basic estimates (e.g. regression coefficient) AND variation (e.g. standard deviation) or associated estimates of uncertainty (e.g. confidence intervals)
☐	☒	For null hypothesis testing, the test statistic (e.g. *F*, *t*, *r*) with confidence intervals, effect sizes, degrees of freedom and *P* value noted<br>*Give P values as exact values whenever suitable.*
☒	☐	For Bayesian analysis, information on the choice of priors and Markov chain Monte Carlo settings
☒	☐	For hierarchical and complex designs, identification of the appropriate level for tests and full reporting of outcomes
☐	☒	Estimates of effect sizes (e.g. Cohen's *d*, Pearson's *r*), indicating how they were calculated

Our web collection on statistics for biologists contains articles on many of the points above.

Software and code

Policy information about availability of computer code

Data collection	no software was used

Data analysis

The plasmidomes of 78 Holstein dairy cows were retrieved from the raw reads of sequenced metagenomes, using the Recycler algorithm. Plasmid-containing 1,3-PD genes were retrieved using local Blast. The plasmid was analysed for the presence of conjugative systems and relaxases using ConjScan of McSyFinder and MOBscan . The raw reads of 311 healthy human individual fecal samples from project SRP100518 were trimmed using Trim Galore , assembled into contigs by Megahit and plasmids were recovered by SCAPP. Raw reads of each of the 311 samples were mapped to plasmid sequence of interest using BBmap, and the plasmid was considered present in a sample if its read coverage was higher than 70% as computed by SAMtools mpileup.
MassLynx and TargetLynx software (v.4.2, Waters) were applied for the acquisition and analysis of data of targeted metabolomics of reuterin compound.
ESV table was created using DADA2 and QIIME.
The sequences from shotgun sequencing were assembled with SPAdes.
The DNA contents of the bacterial strains were compared using Mauve, and Proteinortho.
For STORM microscopy, image acquisition and data reconstruction were performed using the Elyra PS1 system (X100 oil objective) and Zen software (Zeiss).
Quantitive PCR standard curves were obtained using eight dilution points and were calculated using the Rotorgene 6000 series software (Qiagen, Hilden, Germany).
For untargeted metabolomics, chromatogram processing, peak detection and integration was performed using MZmine 2 with signal to noise ratio set to 5 (MZmine-2.41.2). Metabolite annotations were obtained by performing Molecular Networking using the GNPS, by using the Compound Discoverer version 3.3 (Thermo Scientific), by extracting the metabolites present in the KEGG genomes of the bacteria and using MetaboAnalyst.

For manuscripts utilizing custom algorithms or software that are central to the research but not yet described in published literature, software must be made available to editors and reviewers. We strongly encourage code deposition in a community repository (e.g. GitHub). See the Nature Portfolio guidelines for submitting code & software for further information.

Data

Policy information about availability of data

All manuscripts must include a data availability statement. This statement should provide the following information, where applicable:
- Accession codes, unique identifiers, or web links for publicly available datasets
- A description of any restrictions on data availability
- For clinical datasets or third party data, please ensure that the statement adheres to our policy

Genomes sequencing of E.faecalis MM1 and DSM 8630 have been deposited in GenBank (JAMKBU000000000 and JAMKBV000000000). Metabolomics data was deposited to MetaboLights under the identifier MTBLS7248 (www.ebi.ac.uk/metabolights/MTBLS7248).

Research involving human participants, their data, or biological material

Policy information about studies with human participants or human data. See also policy information about sex, gender (identity/presentation), and sexual orientation and race, ethnicity and racism.

Reporting on sex and gender: n/a

Reporting on race, ethnicity, or other socially relevant groupings: n/a

Population characteristics: n/a

Recruitment: n/a

Ethics oversight: n/a

Note that full information on the approval of the study protocol must also be provided in the manuscript.

Field-specific reporting

Please select the one below that is the best fit for your research. If you are not sure, read the appropriate sections before making your selection.

☒ Life sciences ☐ Behavioural & social sciences ☐ Ecological, evolutionary & environmental sciences

For a reference copy of the document with all sections, see nature.com/documents/nr-reporting-summary-flat.pdf

Life sciences study design

All studies must disclose on these points even when the disclosure is negative.

Sample size: all experiments were performed with at least biological triplicates when the biological signal was the most reproducible and up to ten biological replicates in experiments were high deviations were expected

Data exclusions	no data was excluded
Replication	Each experiment was repeated 3 times in triplicates (or more). All attempts in replication were successful.
Randomization	in our experiments we measure the effect of one parameter to another (i.e. enzymatic activity of an enzyme to its substrate ..), thus no randomization was considered
Blinding	no blinding was considered as most of the experiments were performed by one person

Reporting for specific materials, systems and methods

We require information from authors about some types of materials, experimental systems and methods used in many studies. Here, indicate whether each material, system or method listed is relevant to your study. If you are not sure if a list item applies to your research, read the appropriate section before selecting a response.

Materials & experimental systems

n/a	Involved in the study
☒	☐ Antibodies
☒	☐ Eukaryotic cell lines
☒	☐ Palaeontology and archaeology
☒	☐ Animals and other organisms
☒	☐ Clinical data
☒	☐ Dual use research of concern
☒	☐ Plants

Methods

n/a	Involved in the study
☒	☐ ChIP-seq
☒	☐ Flow cytometry
☒	☐ MRI-based neuroimaging
