# Supplementary figures and images for: Plasmid-encoded toxin defence mediates mutualistic microbial interactions
Source: Nat Microbiol. 2023 Dec 27;9(1):108–19. doi: 10.1038/s41564-023-01521-9 (PMC10769881; doi:10.1038/s41564-023-01521-9)

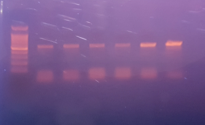

Supplement: Supplementary file 5 — Source data. [file 41564_2023_1521_MOESM5_ESM.zip › Figure_3D_upper_gel.png]

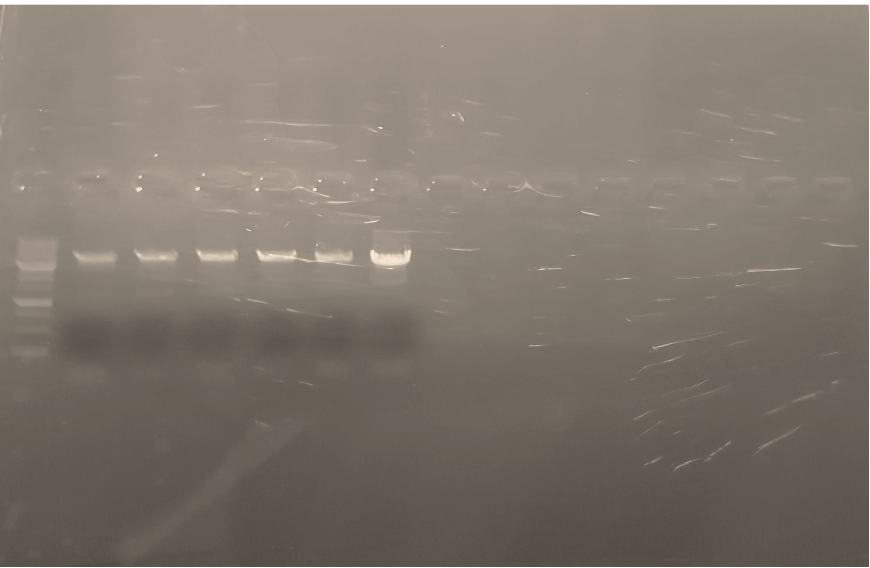

Supplement: Supplementary file 5 — Source data. [file 41564_2023_1521_MOESM5_ESM.zip › Figure_3D_lower_gel.png]

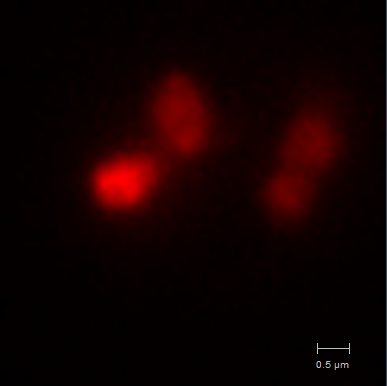

Supplement: Supplementary file 5 — Source data. [file 41564_2023_1521_MOESM5_ESM.zip › Figure_3D_MM1_groel_2_snap-crop.tif]

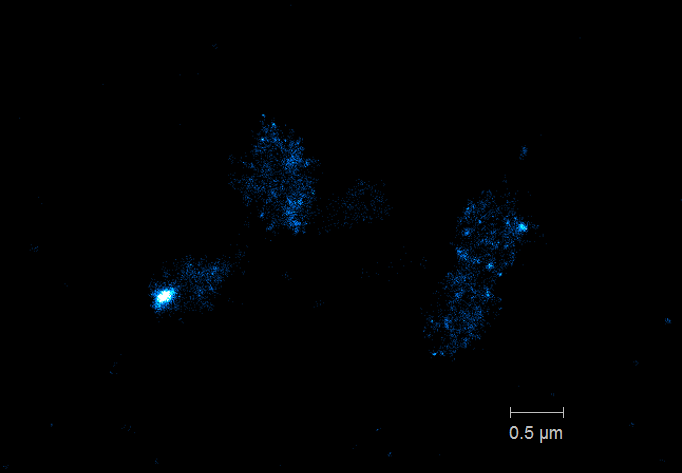

Supplement: Supplementary file 5 — Source data. [file 41564_2023_1521_MOESM5_ESM.zip › Figure_3D_MM1_groel_2_storm_PALM.tif]

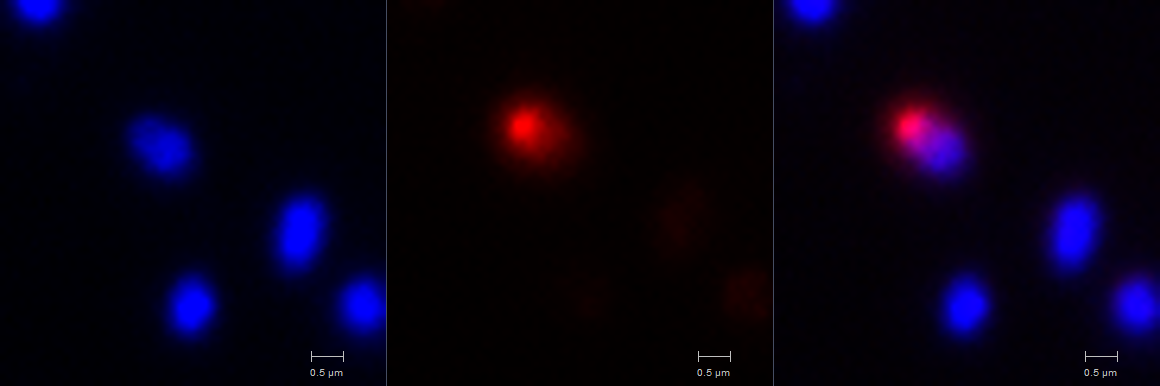

Supplement: Supplementary file 5 — Source data. [file 41564_2023_1521_MOESM5_ESM.zip › Figure_3D_MM1_groel_3_snap.tif]

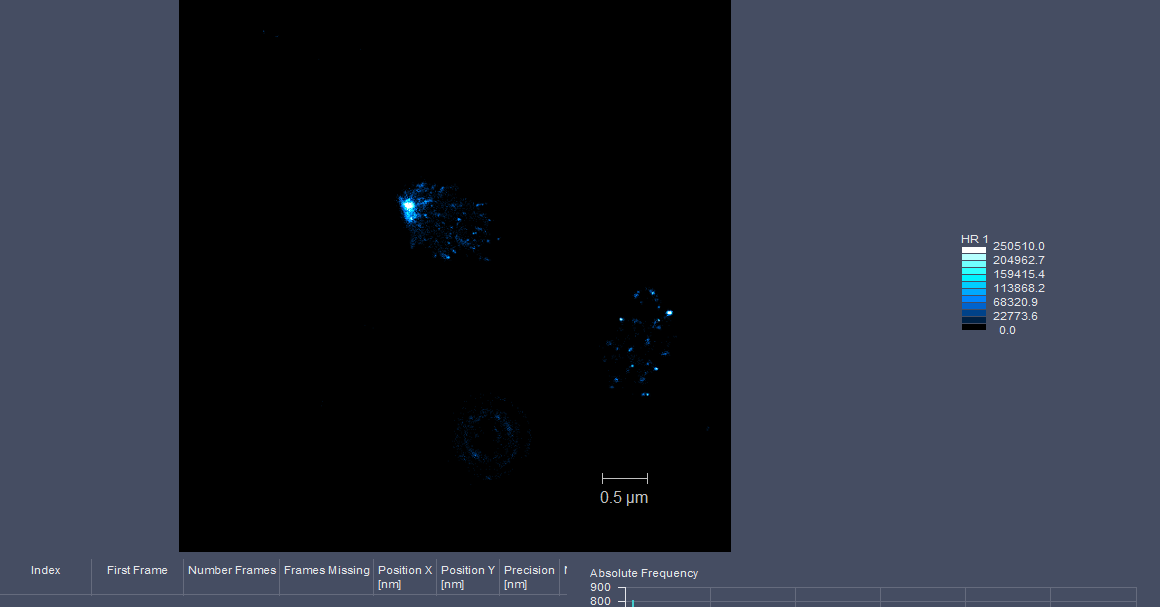

Supplement: Supplementary file 5 — Source data. [file 41564_2023_1521_MOESM5_ESM.zip › Figure_3D_MM1_groel_3._stormczi_PALM_.tif]

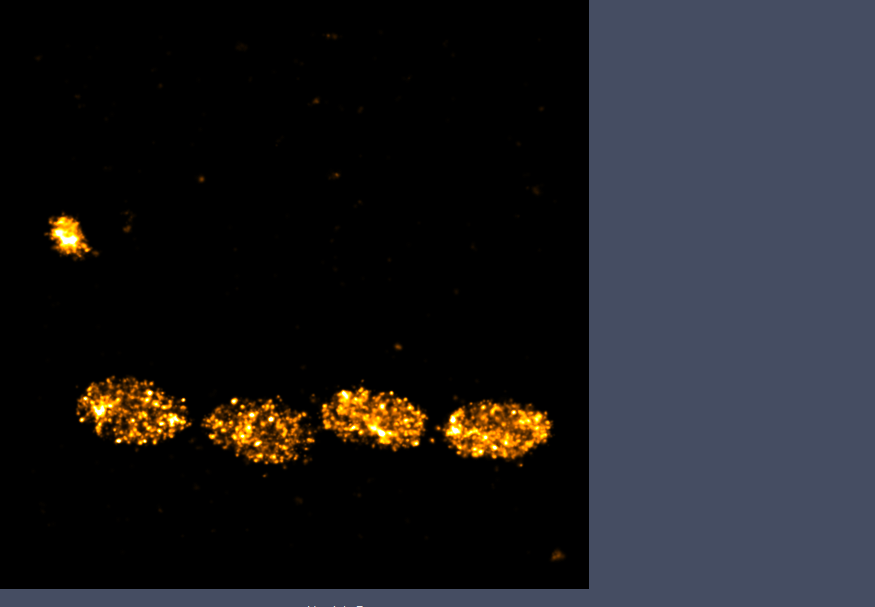

Supplement: Supplementary file 5 — Source data. [file 41564_2023_1521_MOESM5_ESM.zip › Figure_3D_MM1_plasmid_snap_5_storm_PALM_orange_2.tif]

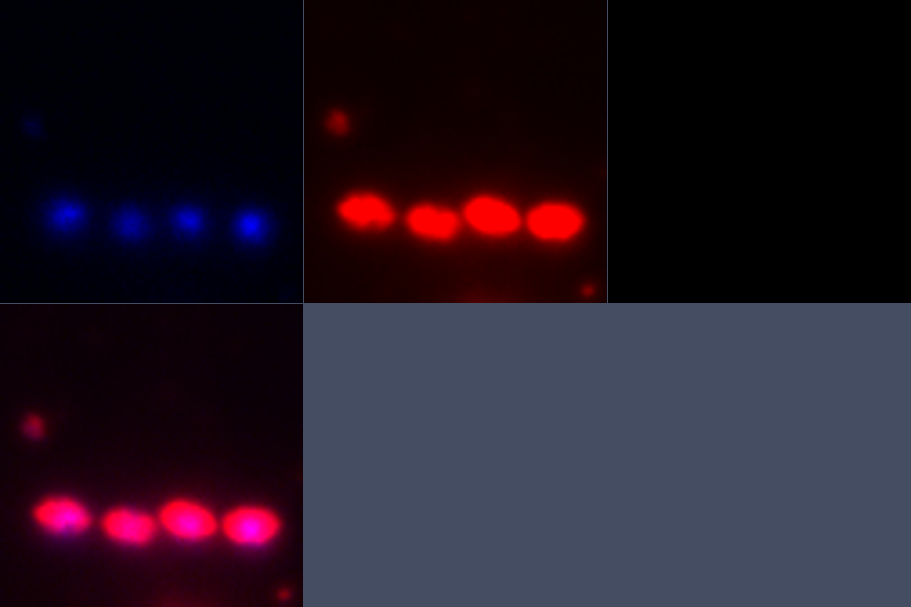

Supplement: Supplementary file 5 — Source data. [file 41564_2023_1521_MOESM5_ESM.zip › Figure_3D_MM1_plasmid_snap_5-2.tif]

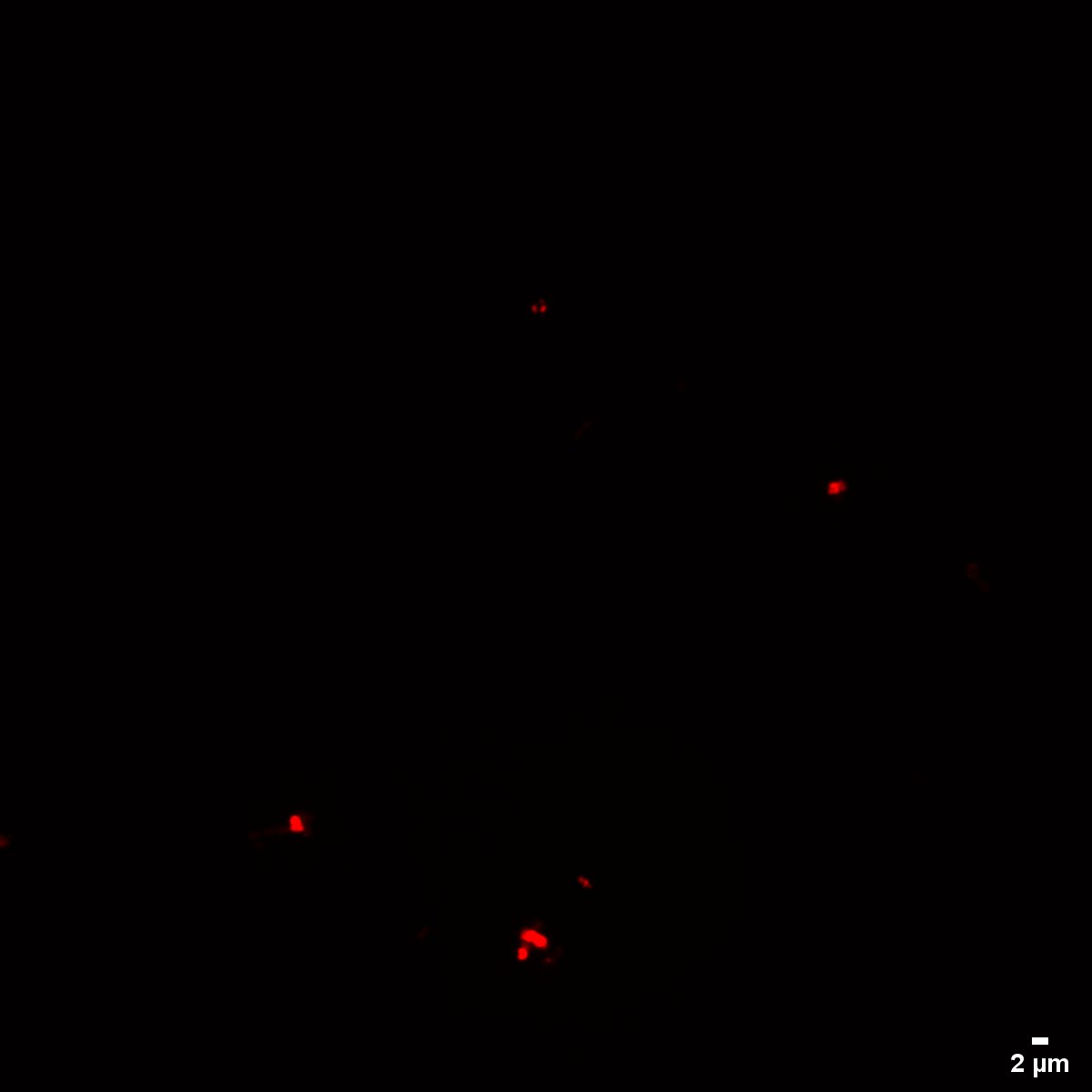

Supplement: Supplementary file 9 — Source image. [file 41564_2023_1521_MOESM9_ESM.zip › Extended_Data_figure_4__MM1_probe_middle.jpg]

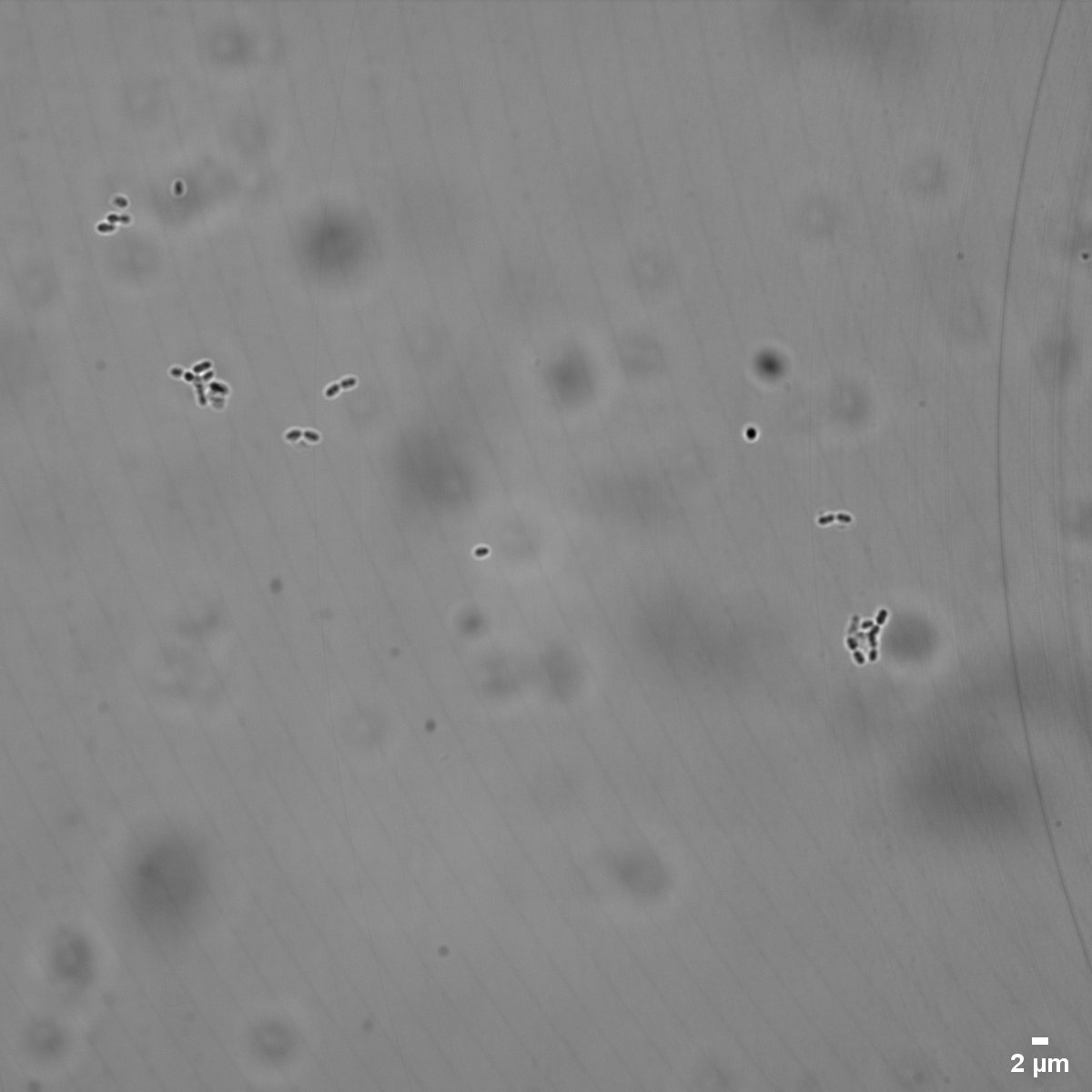

Supplement: Supplementary file 9 — Source image. [file 41564_2023_1521_MOESM9_ESM.zip › Extended_Data_figure_4__MM1-probe_up-bright.jpg]

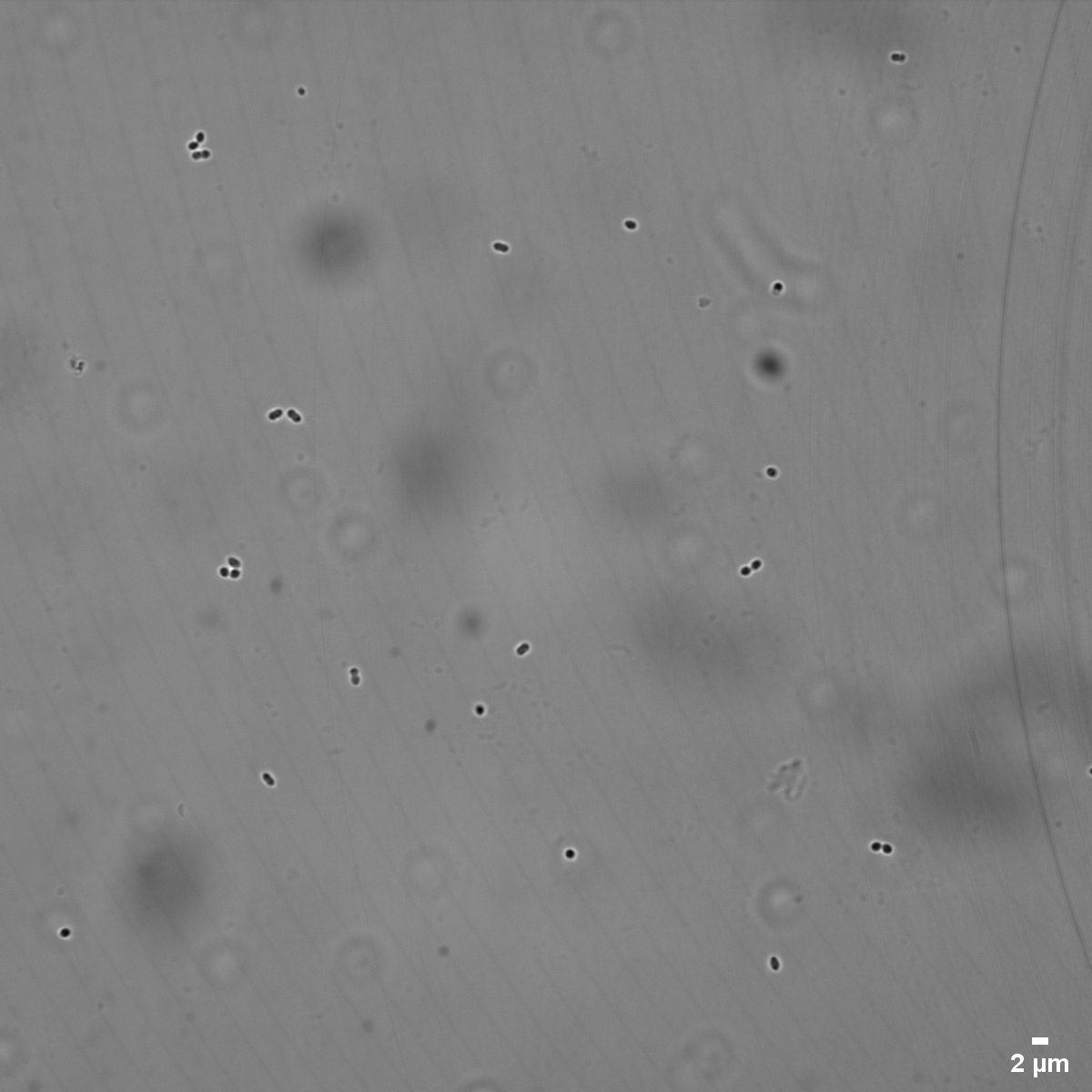

Supplement: Supplementary file 9 — Source image. [file 41564_2023_1521_MOESM9_ESM.zip › Extended_Data_figure_4_8630-probe_middle-bright.jpg]

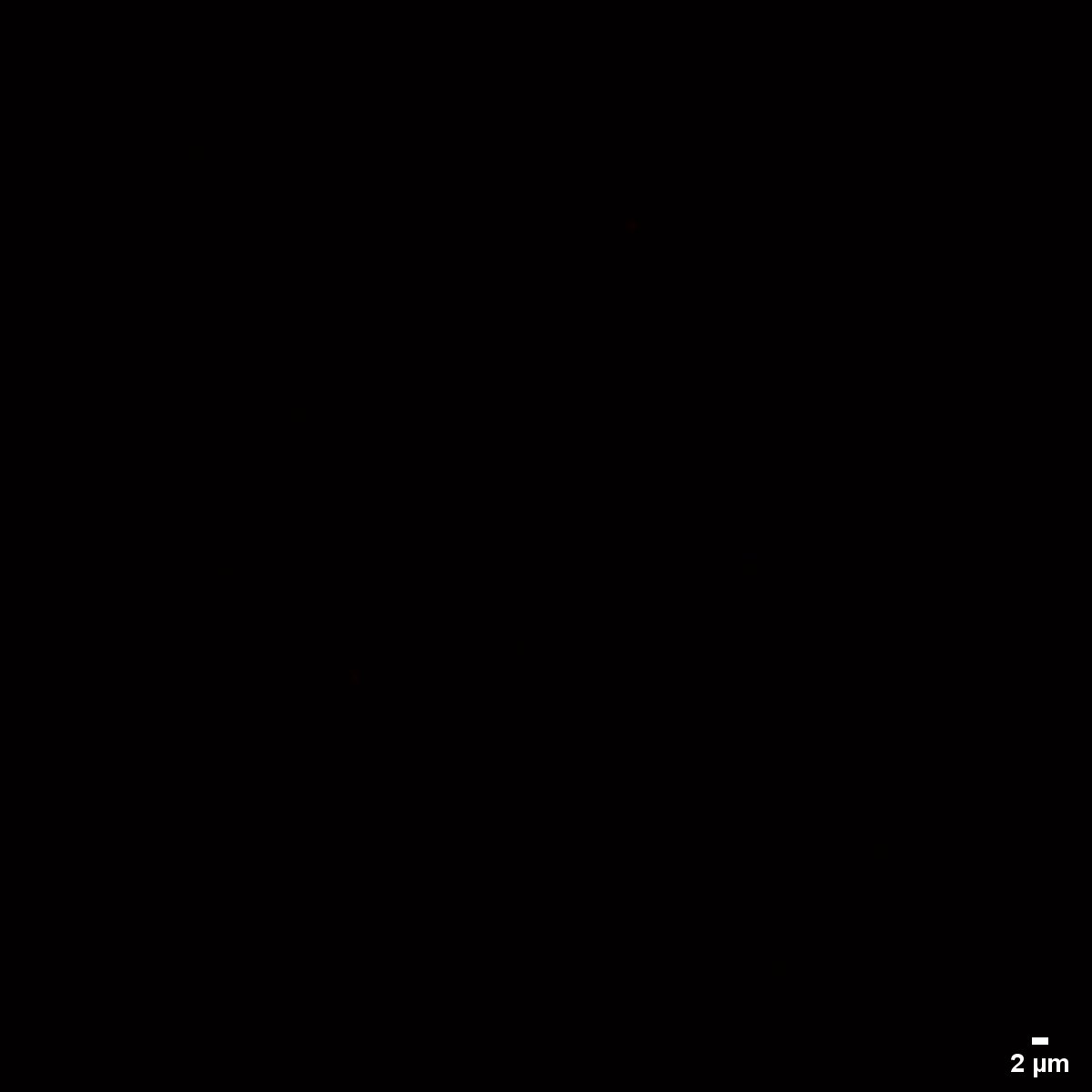

Supplement: Supplementary file 9 — Source image. [file 41564_2023_1521_MOESM9_ESM.zip › Extended_Data_figure_4_8630-probe_middle.jpg]

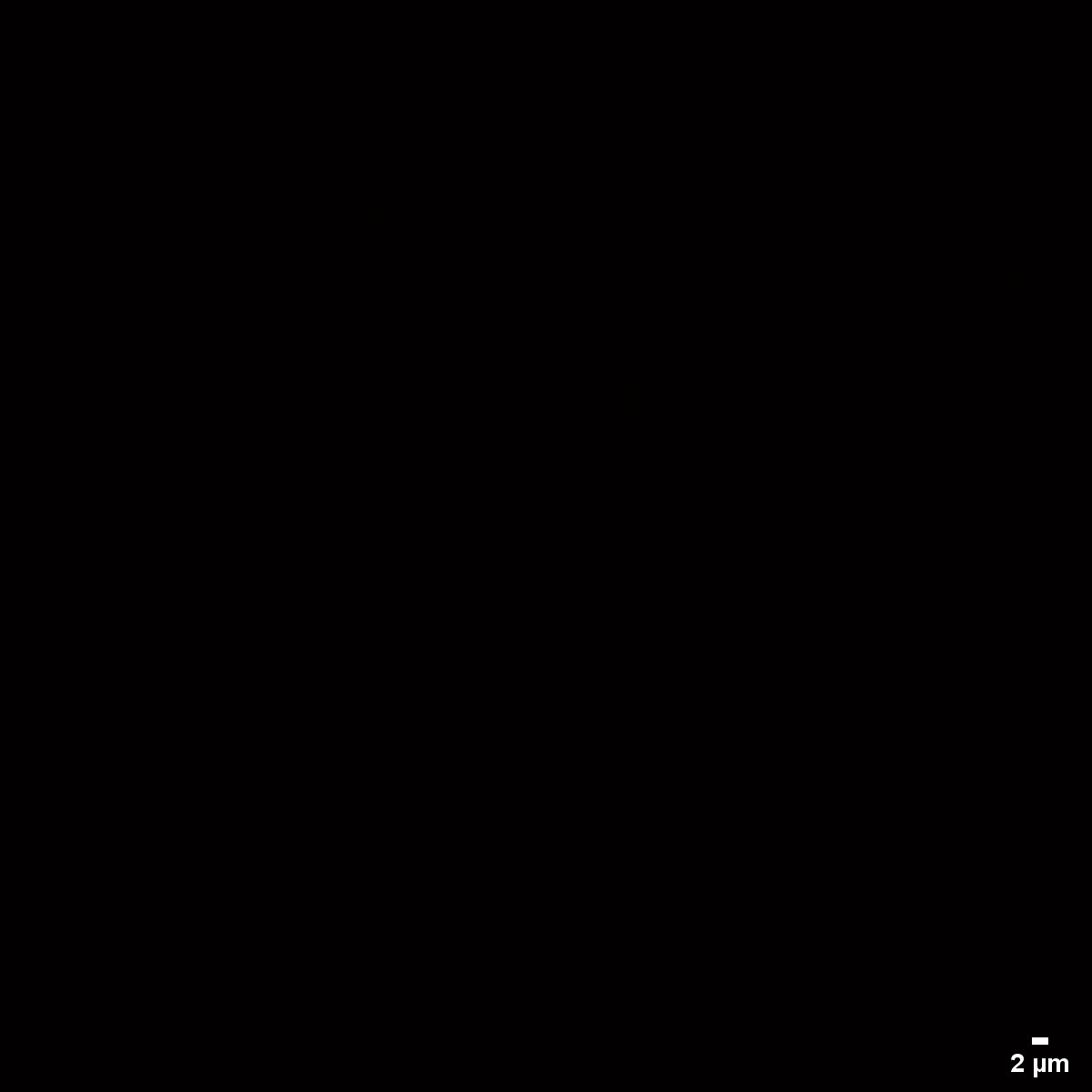

Supplement: Supplementary file 9 — Source image. [file 41564_2023_1521_MOESM9_ESM.zip › Extended_Data_figure_4_8630-probe_up_.jpg]

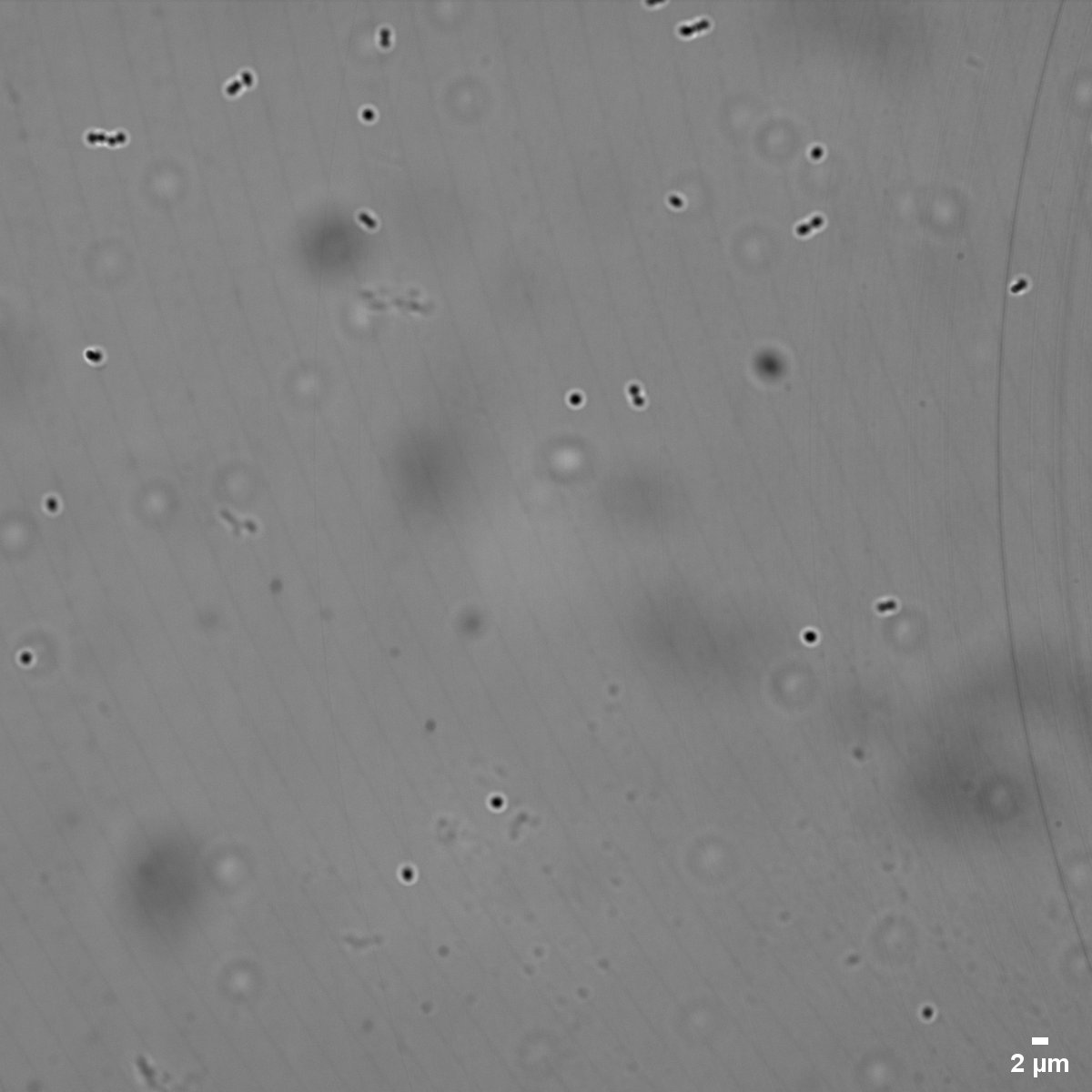

Supplement: Supplementary file 9 — Source image. [file 41564_2023_1521_MOESM9_ESM.zip › Extended_Data_figure_4_8630-probe_up-bright.jpg]

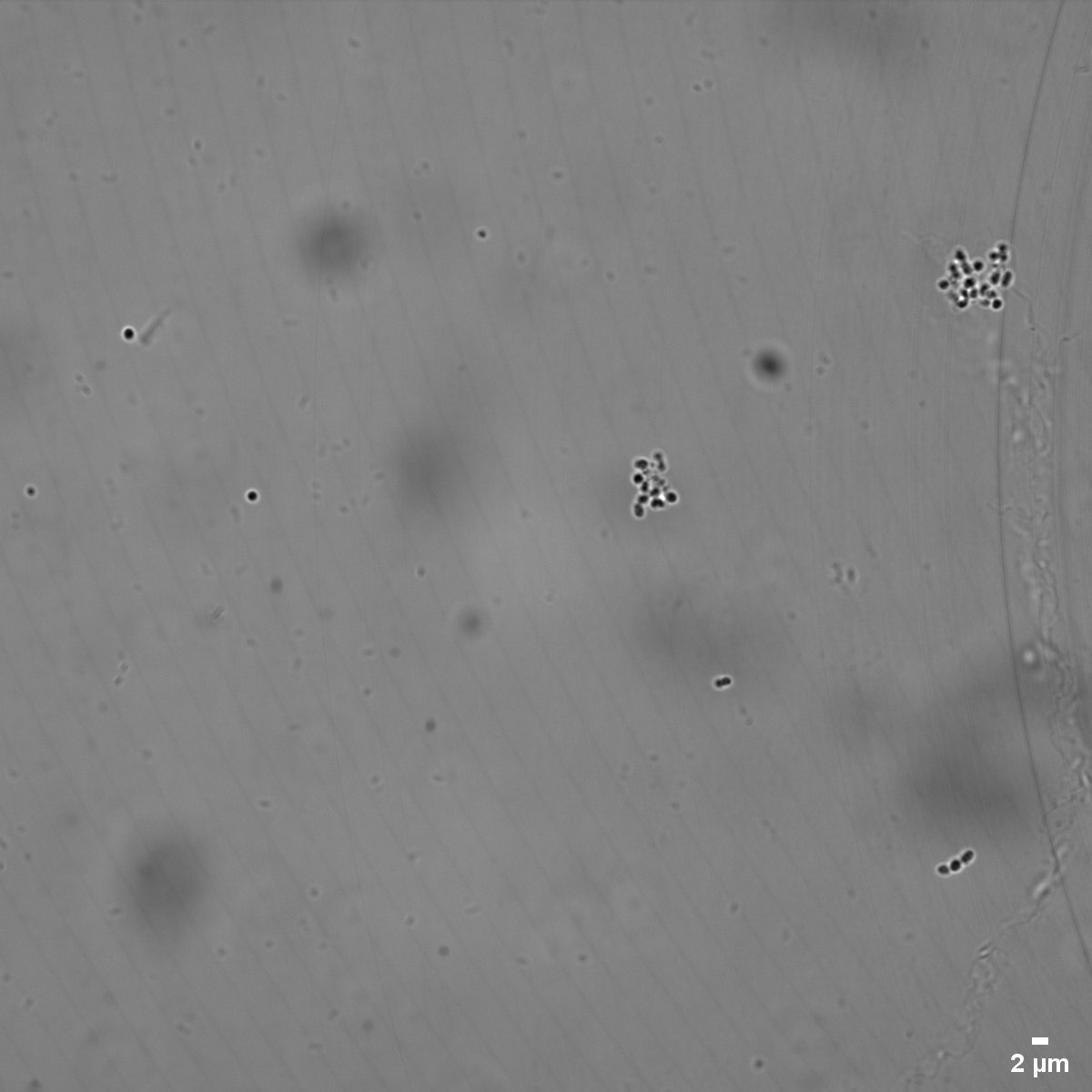

Supplement: Supplementary file 9 — Source image. [file 41564_2023_1521_MOESM9_ESM.zip › Extended_Data_figure_4_8630no-probe-bright.jpg]

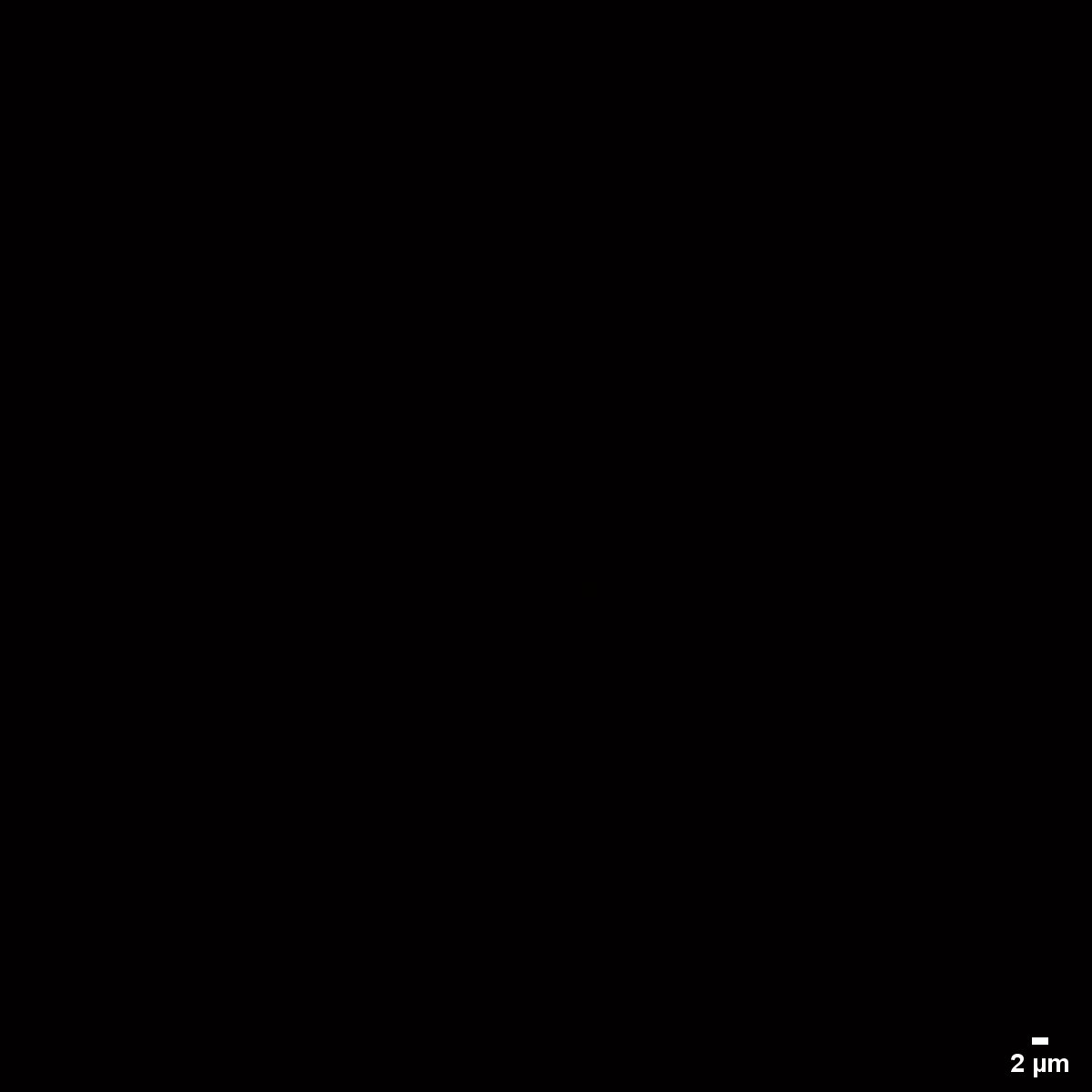

Supplement: Supplementary file 9 — Source image. [file 41564_2023_1521_MOESM9_ESM.zip › Extended_Data_figure_4_8630no-probe.jpg]

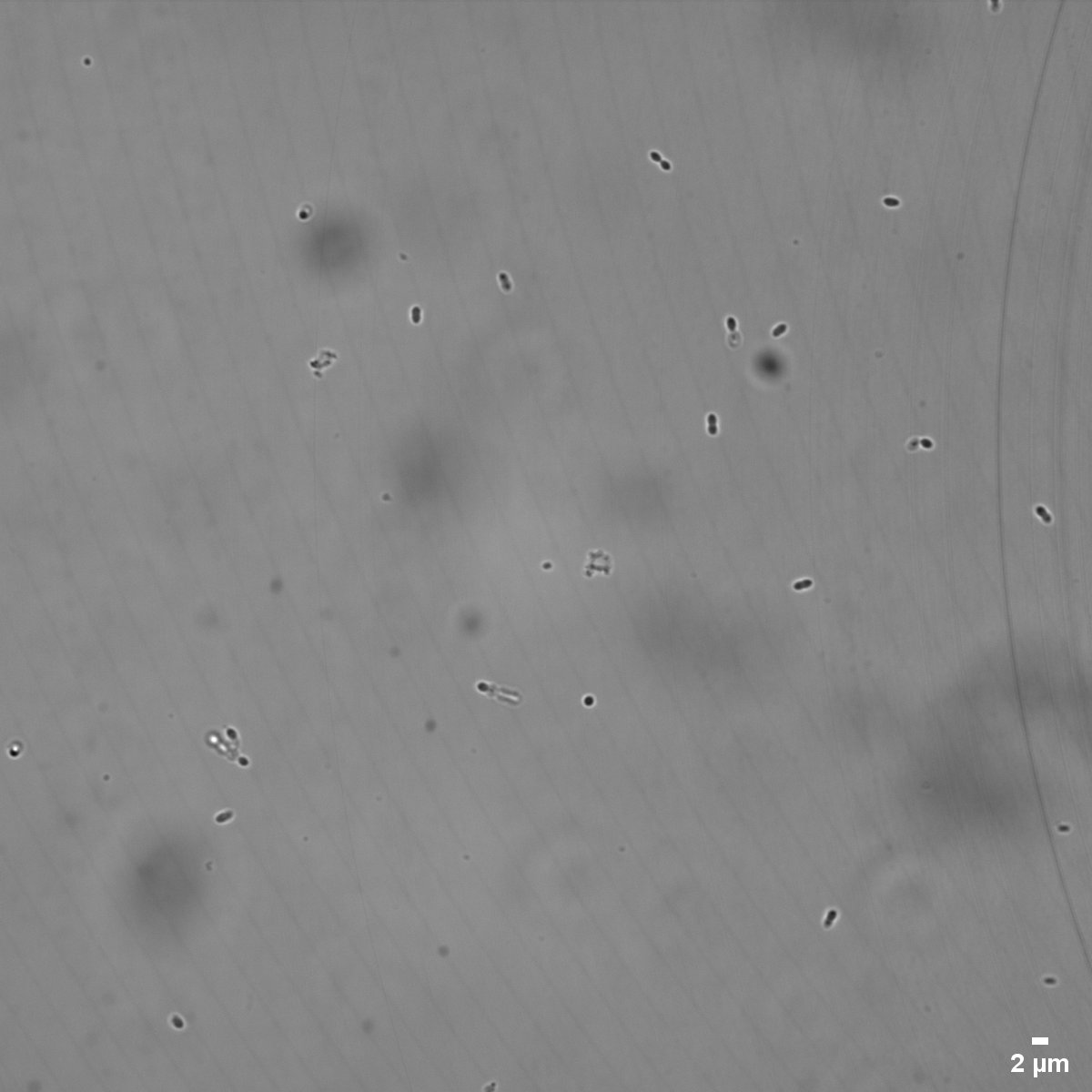

Supplement: Supplementary file 9 — Source image. [file 41564_2023_1521_MOESM9_ESM.zip › Extended_Data_figure_4_MM1_noprobe-bright.jpg]

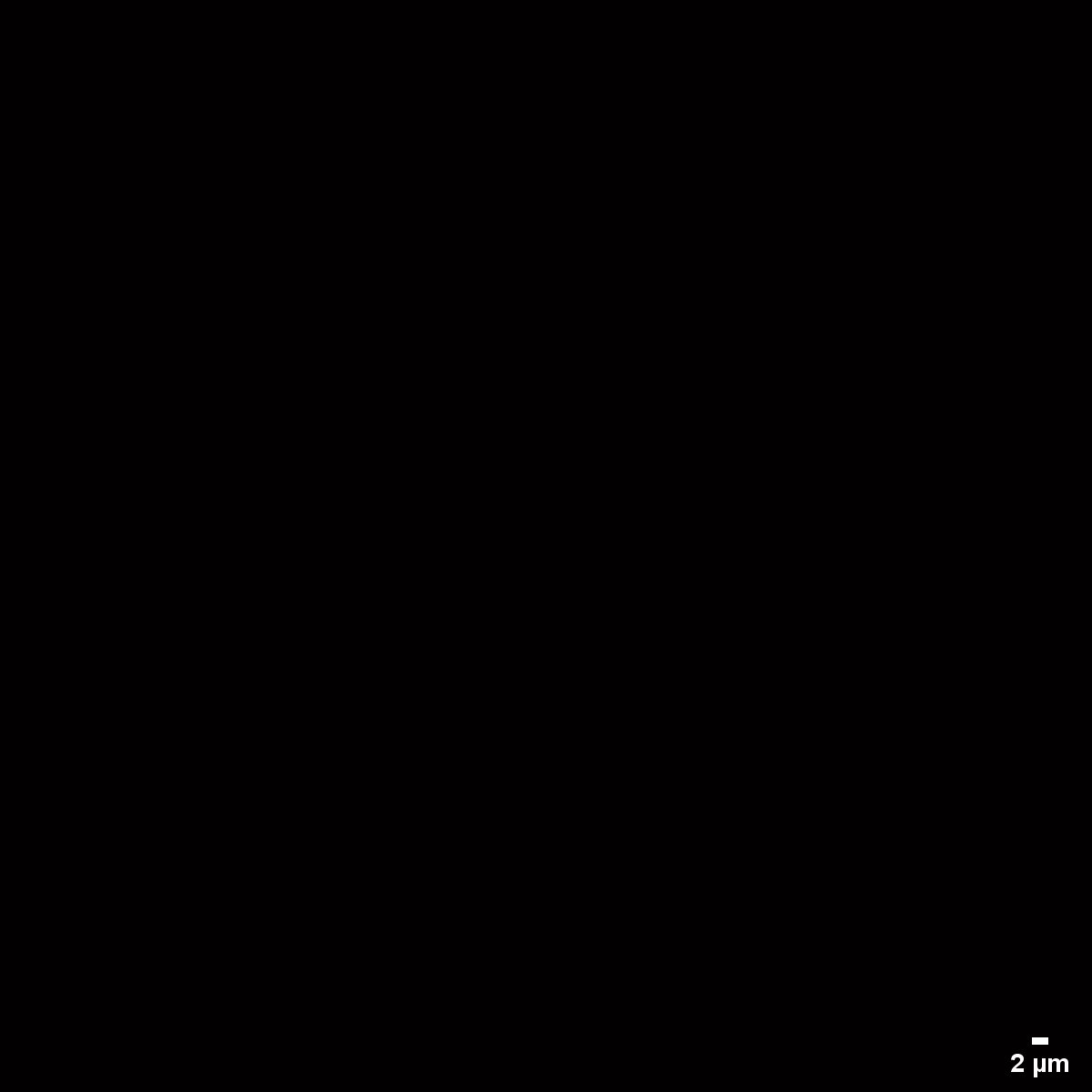

Supplement: Supplementary file 9 — Source image. [file 41564_2023_1521_MOESM9_ESM.zip › Extended_Data_figure_4_MM1_noprobe.jpg]

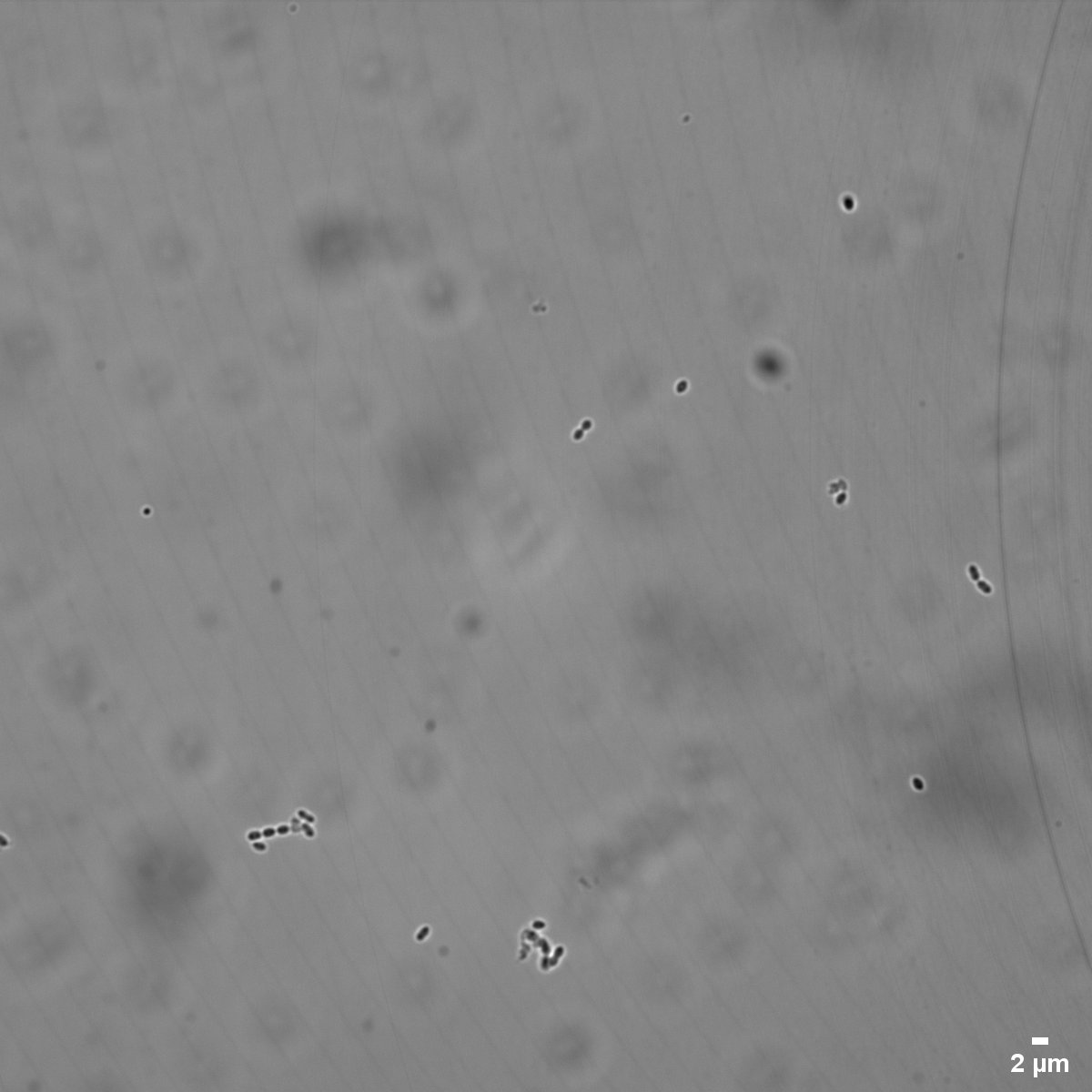

Supplement: Supplementary file 9 — Source image. [file 41564_2023_1521_MOESM9_ESM.zip › Extended_Data_figure_4_MM1_probe_middle-bright.jpg]

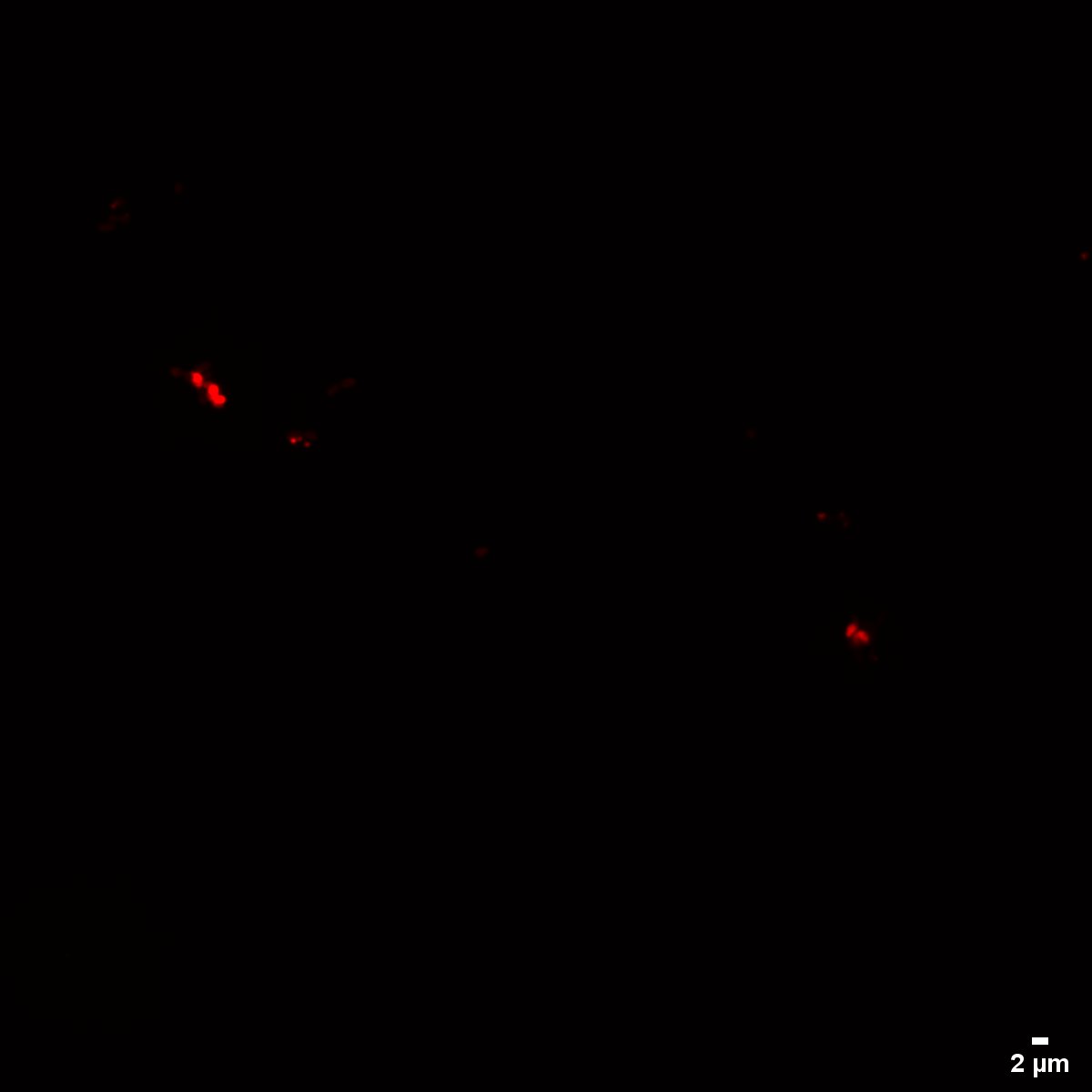

Supplement: Supplementary file 9 — Source image. [file 41564_2023_1521_MOESM9_ESM.zip › Extended_Data_figure_4_nMM1_probe_up.jpg]
